# Defective viral genomes of porcine reproductive and respiratory syndrome virus type 2: Generation, characterization and interference effects on viral replication

**DOI:** 10.1371/journal.ppat.1014755

**Published:** 2026-10-28

**Authors:** Hao Song, Guoqing Liu, Yongbo Yang, Meng Chen, Haojie Wang, Zeqing Liu, Xinyi Huang, Tongqing An

**Affiliations:** 1 State Key Laboratory of Animal Disease Control and Prevention, Harbin Veterinary Research Institute, Chinese Academy of Agricultural Sciences, Harbin, China; 2 Heilongjiang Provincial Key Laboratory of Veterinary Immunology, Harbin Veterinary Research Institute, Chinese Academy of Agricultural Sciences, Harbin, China; TWINCORE Center of Experimental and Clinical Infection Research: TWINCORE Zentrum fur Experimentelle und Klinische Infektionsforschung GmbH, GERMANY

## Abstract

Porcine reproductive and respiratory syndrome virus type 2 (PRRSV-2) is a major swine pathogen that causes substantial economic losses worldwide. Defective viral genomes (DVGs) are important regulators of viral replication, persistence, and evolution in many RNA viruses. However, the characteristics and functions of DVGs generated during PRRSV-2 infection remain unknown. Here, we systematically analyzed publicly available high-throughput sequencing datasets and characterized DVGs generated during both *in vitro* and *in vivo* PRRSV-2 infection. We found that PRRSV-2 preferentially generates large deletion DVGs, particularly in porcine alveolar macrophages (PAMs), and that DVG formation is associated with microhomology sequences, RNA secondary structure and AU-rich sequences. Break positions were enriched in the GP5/M genomic region, whereas rejoin positions clustered predominantly within the nsp1/nsp2 genomic region of the PRRSV-2 genome. Notably, the deletion events within the same gene were highly enriched in the nsp2 region, where multiple naturally occurring nsp2 deletion sites overlapped with DVG junction sites. These findings suggest that DVG-associated recombination may contribute to the formation of PRRSV-2 nsp2 deletion variants. Functionally, PRRSV-2-derived DVGs exhibited potent broad-spectrum antiviral activity against multiple prevalent PRRSV-2 lineage strains and PRRSV-1. Overall, our findings provide the first systematic characterization of PRRSV-2 DVGs, reveal a potential evolutionary link between DVGs and nsp2 deletion variants, and highlight the antiviral potential of PRRSV-2 DVGs.

## Author summary

Porcine reproductive and respiratory syndrome virus type 2 (PRRSV-2) remains one of the most economically devastating pathogens affecting the global swine industry, yet the characteristics and biological significance of defective viral genomes (DVGs) generated during infection remain unclear. In this study, we provide the first systematic characterization of PRRSV-2 DVGs generated during both *in vitro* and *in vivo* infection. We found that PRRSV-2 preferentially produces large deletion DVGs in its natural target cells, porcine alveolar macrophages, and that DVG formation is strongly associated with microhomology sequences, RNA secondary structure and AU-rich regions. Notably, our analyses suggest a potential link between DVG-associated recombination and the emergence of naturally occurring nsp2 deletion variants in PRRSV-2. In addition, representative PRRSV-2-derived DVGs exhibited broad antiviral activity against multiple PRRSV-2 lineages as well as PRRSV-1 strains. Overall, these findings reveal the characteristics and functions of DVGs generated during PRRSV-2 infection and provide new insights into PRRSV-2 DVG research and the development of novel antiviral drugs against PRRSV-2.

## Introduction

Porcine reproductive and respiratory syndrome (PRRS) is a major viral disease affecting the swine industry worldwide, characterized by reproductive failure in sows and respiratory disorders in piglets and adult pigs, resulting in substantial economic losses to global pork production [1–3]. The causative agent, PRRS virus (PRRSV), is an enveloped, single-stranded positive-sense RNA virus with a genome of approximately 15 kb that encodes at least ten open reading frames (ORFs): ORF1a, ORF1b, ORF2a, ORF2b, ORF3, ORF4, ORF5, ORF5a, ORF6, and ORF7 [4–6]. PRRSV belongs to the family *Arteriviridae* within the order *Nidovirales* and can be divided into two species: *Betaarterivirus suid* 1 (PRRSV-1, formerly known as European-type PRRSV) and *Betaarterivirus suid* 2 (PRRSV-2, formerly known as North American-type PRRSV) [7–9]. Furthermore, PRRSV-2 can be classified into 11 distinct lineages, with the predominant strains circulating in China including lineage 1 (NADC30-like and NADC34-like), lineage 3 (QYYZ-like), lineage 5 (VR-2332-like), and lineage 8 (CH-1a-like and JXA1-like) [10,11]. Highly pathogenic PRRSV-2 (HP-PRRSV), belonging to lineage 8.7 of PRRSV-2, emerged in China, Thailand, and Vietnam in 2006 and has since become the dominant PRRSV-2 lineage in China and some other Asian countries [12,13]. Lineage 1, first described in 2013, includes L1.8 (NADC30-like PRRSV-2), which has become widely prevalent in China, and L1.5 (NADC34-like PRRSV-2), which has been increasingly reported in pig populations in recent years [14,15]. The high mutation rate, frequent recombination events, and pronounced immune evasion strategies of PRRSV-2 have made its control and vaccine development particularly challenging [3,16–18].

The genetic diversity of PRRSV-2 is fundamentally driven by the error-prone nature of its viral RNA-dependent RNA polymerase (RdRp) [19]. Owing to the lack of proofreading activity of RdRp, viral RNA replication is prone to errors and template switching, thereby contributing to the genetic mutations and recombination commonly observed in RNA viruses [20,21]. This process also gives rise to aberrant replication products known as defective viral genomes (DVGs), which typically contain large internal deletions in the viral genome and therefore lose the ability to complete an independent replication cycle [22,23]. Accumulating evidence indicates that DVGs are widely generated during infections by some RNA viruses and play a critical role in viral pathogenesis [24,25]. When RNA viruses are serially passaged at a high multiplicity of infection (MOI), DVGs typically accumulate within viral populations, with changes in their diversity and composition [24,26–28]. Based on their structural features, DVGs can be categorized into two types: deletion-type DVGs and copy-back DVGs [25]. Deletion-type DVGs retain the essential promoter regions and *cis*-acting elements at the 5′and 3′ genomic termini that are required for replication and packaging, while harboring extensive internal deletions, and are commonly observed in coronaviruses, reoviruses and influenza viruses [25,28–30]. In contrast, copy-back DVGs contain a portion of the genome flanked by either the 3′ or 5′ UTR, and its reverse complement, which have been found in many non-segmented negative-sense RNA viruses, such as respiratory syncytial virus, paramyxoviruses, and filoviruses [31–33]. Functionally, DVGs can interfere with the replication of wild-type (WT) virus by competing for viral polymerase and cellular resources, thereby limiting the replication of the full-length genome [34–36]. In addition, they can act as potent pathogen-associated molecular patterns (PAMPs) that are recognized by host pattern recognition receptors such as RIG-I and MDA5, leading to the activation of interferons and interferon-stimulated genes (ISGs) and enhancing the host antiviral response [37–41]. In several RNA viruses, including respiratory syncytial virus, measles virus and Sendai virus, DVGs have been related to viral persistence, immune modulation, and antiviral protection [31,42,43].

The generation of defective interfering RNA during PRRSV-2 infection has been reported [44–46]. However, previous studies have primarily focused on the identification and validation of individual DVG events and have not systematically characterized the genome-wide features and formation patterns of PRRSV-2 DVGs. In addition, these studies relied on RT-PCR to identify DVGs, which limited a comprehensive assessment of the diversity, distribution, and abundance of PRRSV-2 DVGs. With the development of high-throughput sequencing technologies and the establishment of multiple DVG-detection algorithms [47–49], new tools will help to comprehensively reveal the characteristics of PRRSV-2 DVGs.

Here, we systematically characterized PRRSV-2 DVGs generated during infection of pig lung tissues, porcine alveolar macrophages (PAMs), and Marc-145 cells. We demonstrated that DVGs are widely generated during PRRSV-2 infection and comprehensively analyzed their deletion characteristics and sequence features associated with DVG formation. In addition, we found a potential relationship between DVG generation and naturally occurring PRRSV-2 nsp2 deletion variants. Finally, we evaluated the effects of representative DVGs on viral replication. Together, these findings provide a comprehensive view of PRRSV-2 DVGs and improve our understanding of their generation, diversity, and biological significance during PRRSV-2 infection.

## Results

### DVGs are ubiquitously produced during PRRSV-2 infection *in vitro* or *in vivo*

To investigate the characteristics of PRRSV-2 DVG formation, we surveyed available PRRSV-2 RNA-seq datasets in the NCBI database and selected 24 samples that met the selection criteria, including 17 cell samples (9 PAM samples and 8 Marc-145 cell samples) and 7 *in vivo* tissue samples. These samples encompassed four PRRSV-2 lineages, including 15 samples from lineage 8, 4 from lineage 1, 4 from lineage 5, and 1 from lineage 3. Sequencing reads were analyzed using the ViReMa pipeline to identify recombination junction sites. The depth of sequence coverage indicated that all 24 datasets had sufficient sequencing quality for DVG analyses (Fig 1A–1C). Given the differences in sequencing depth, MOI, and experimental conditions across datasets, DVG levels were normalized to viral read counts to facilitate visualization of DVG abundance within individual samples. DVGs were detected in all analyzed cell-derived datasets (Fig 1D and 1E) and in all tissue-derived datasets (Fig 1F), with reads ranging from dozens to tens of thousands (S1 Table). Among the tissue-derived datasets, BALF_Li11 originated from bronchoalveolar lavage fluid (BALF) cells from infected pigs, whereas the remaining samples were derived from lung tissue. In addition, because the Lung_S022 and Lung_G128 samples contained relatively few DVGs, they were excluded from subsequent analyses of DVG characteristics to ensure the accuracy of the analysis. Together, these results indicate that DVGs are generated during PRRSV-2 infection in both *in vitro* and *in vivo.*

**Fig 1:**
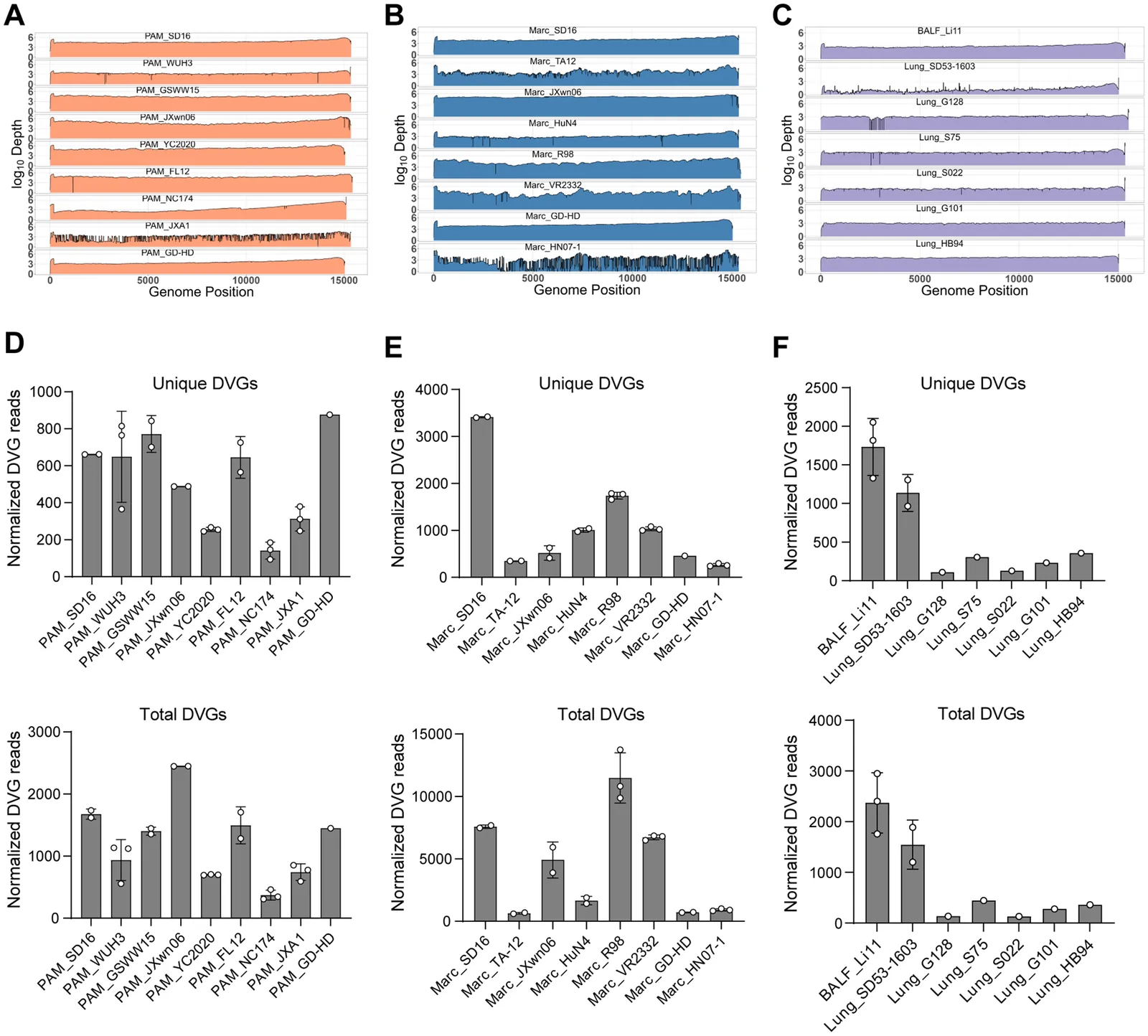
Quantitative analysis of DVGs in PRRSV-2 *in vitro* and *in vivo* infection samples. **(A–C)** RNA-seq depth of each PRRSV-2 strain obtained from PAMs (A) and Marc-145 cells (B) and lung tissue (C) samples. **(D–F)** Normalized abundance of unique and total DVGs identified from PAMs **(D)**, Marc-145 cells **(E)**, and lung tissues **(F)**. Both +sense and −sense DVG reads were included, and the numbers of unique and total DVG reads were normalized per 10⁶ viral reads. Each point represents an individual biological replicate, and error bars indicate the mean ± SD across biological replicates within each dataset.

### PRRSV-2 infection in PAMs preferentially generates DVGs with large deletion

During the analysis, we found that the deletion lengths of PRRSV-2 DVGs exhibited pronounced heterogeneity. To determine whether deletion length is influenced by viral strain or host cell type, we compared the DVG deletion length across samples. Kernel density analyses of DVG deletion lengths from PAMs revealed a consistent bimodal pattern across strains, with a dominant peak at 10,000–15,000 nucleotide (nt) (Fig 2A and S1A Fig). In contrast, although the deletion length distribution of DVGs from Marc-145 cells (Fig 2B and S1B Fig) and lung tissue (Fig 2C and S1C Fig) also exhibited bimodality, the relative peak heights varied among strains. These findings suggested that PAMs may be more permissive for the generation of large deletion DVGs. To validate this observation, we performed a detailed kernel density analysis of DVG deletion lengths specifically in PAMs. The distribution displayed two prominent peaks: one at 0–2000 nt and another at 10,000–15,000 nt, with a clear trough near 4000 nt (Fig 2D). Based on this inflection point, we defined deletions <4000 nt as small deletion DVGs and those ≥4000 nt as large deletion DVGs. We then quantified the relative proportions of large and small deletion DVGs across datasets derived from PAMs, Marc-145 cells, and lung tissues. PAM samples (n = 9) exhibited a higher proportion of large deletion, suggesting that PRRSV-2 infection in PAMs tends to generate large deletion DVGs (Fig 2E). In contrast, Marc-145 cells (n = 8) and lung tissue (n = 5) samples displayed greater variability in the composition of deletion sizes. Furthermore, we quantified the relative proportions of large and small deletions within their cell type. Consistent with our earlier observations, large deletion DVGs were more abundant than small deletion DVGs in PAM samples (Fig 2F). In contrast, there was no difference in the proportion of large deletions and small deletions between samples derived from Marc-145 cells and lung tissue. Similarly, we analyzed DVG size and classified DVGs shorter than 6000 nt as short DVGs (S2A Fig). Notably, because DVG size and DVG deletion length are both calculated from the genomic coordinates of the DVG junctions and therefore represent different ways of describing the same coordinate information, the proportion of short DVGs was expected to be higher than that of long DVGs in PAMs. Consistent with this expectation, the proportion of short DVGs was significantly higher than that of long DVGs in PAMs, whereas no significant difference was observed between short and long DVGs in Marc-145 cells or lung tissue (S2B and S2C Fig).

**Fig 2:**
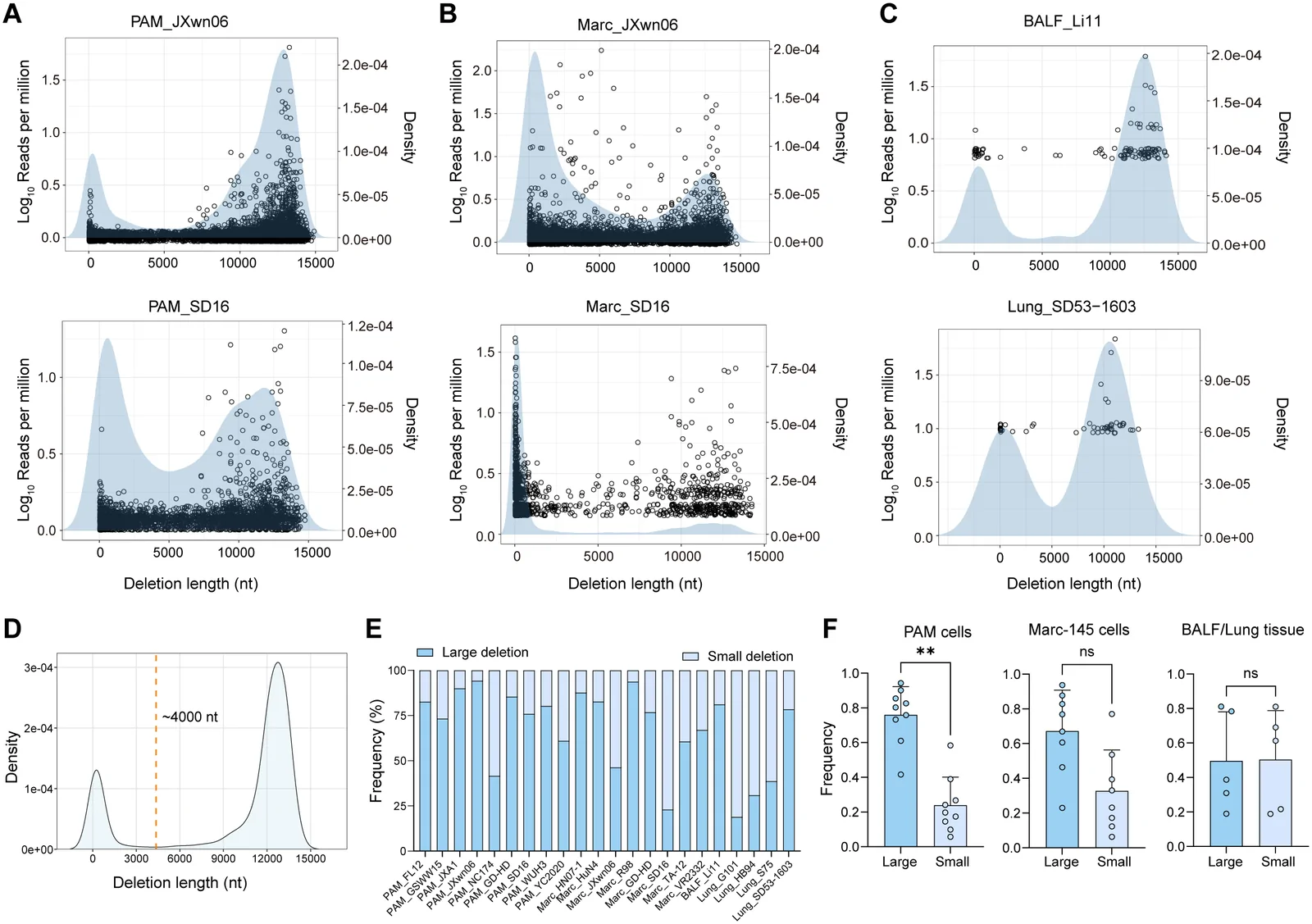
Analysis of the deletion length of PRRSV-2 DVGs. **(A–C)** Distribution of DVG deletion lengths in PAM cells **(A)**, Marc-145 cells **(B)**, and BALF/lung **(C)** samples from different PRRSV-2 strains. Each point represents the number of reads supporting an individual deletion. Reads per million represent the number of DVG events per million viral reads. Kernel density curves were generated using non-parametric kernel density estimation with the density() function in R and overlaid to illustrate the distribution of deletion lengths for each strain. The extension of the x-axis in the KDE curves to negative deletion lengths is a smoothing artifact and has no biological significance. **(D)** Pooled kernel density estimation of DVG deletion lengths, weighted by DVG counts across all samples. The local minimum was used as the threshold distinguishing small deletion DVGs from large deletion DVGs. **(E)** Proportion of small deletion and large deletion DVGs produced by each PRRSV-2 strain in cells or tissue, based on the data-driven threshold identified in panel **D. (F)** Comparison of the relative abundance of small deletion and large deletion DVGs within PAM cells, Marc-145 cells, and lung tissue. Error bars represent the mean ± SD. Statistical significance was assessed using a two-tailed paired Student’s t-test. ns, not significant; **, *P* < 0.01.

### DVG junctions are enriched in the nsp1/nsp2 and GP5/M genomic regions

The above analysis revealed a preference for specific deletion lengths among PRRSV-2 DVGs, but the genomic distribution of their break and rejoin positions remained unclear. Therefore, DVG break and rejoin positions were mapped onto the PRRSV-2 genome to characterize their spatial distribution. Although both analyses were based on the same DVG junction events, they describe distinct features of DVG formation: deletion length and genomic location. Mapping of DVG spatial distribution revealed that break positions were primarily concentrated between 10,000–15,000 nt, whereas rejoin positions clustered between 0–5000 nt. This spatial distribution was consistent with the predominance of large deletion DVGs observed above and resulted in a prominent concentration of DVG junctions in the corresponding region of the plot (Fig 3A and S3 Fig). Notably, in the Marc_JXwn06 sample, DVGs not only concentrated in the upper-left region but also showed additional clustering in surrounding areas. All samples also exhibited a minor clustering of DVGs near the diagonal toward the upper-right region of the plot, corresponding primarily to small deletion DVGs located near the 3′ end of the genome (Fig 3A). These results indicate that high-frequency DVGs in PRRSV-2 are not randomly distributed across the genome but exhibit distinct spatial preferences.

**Fig 3:**
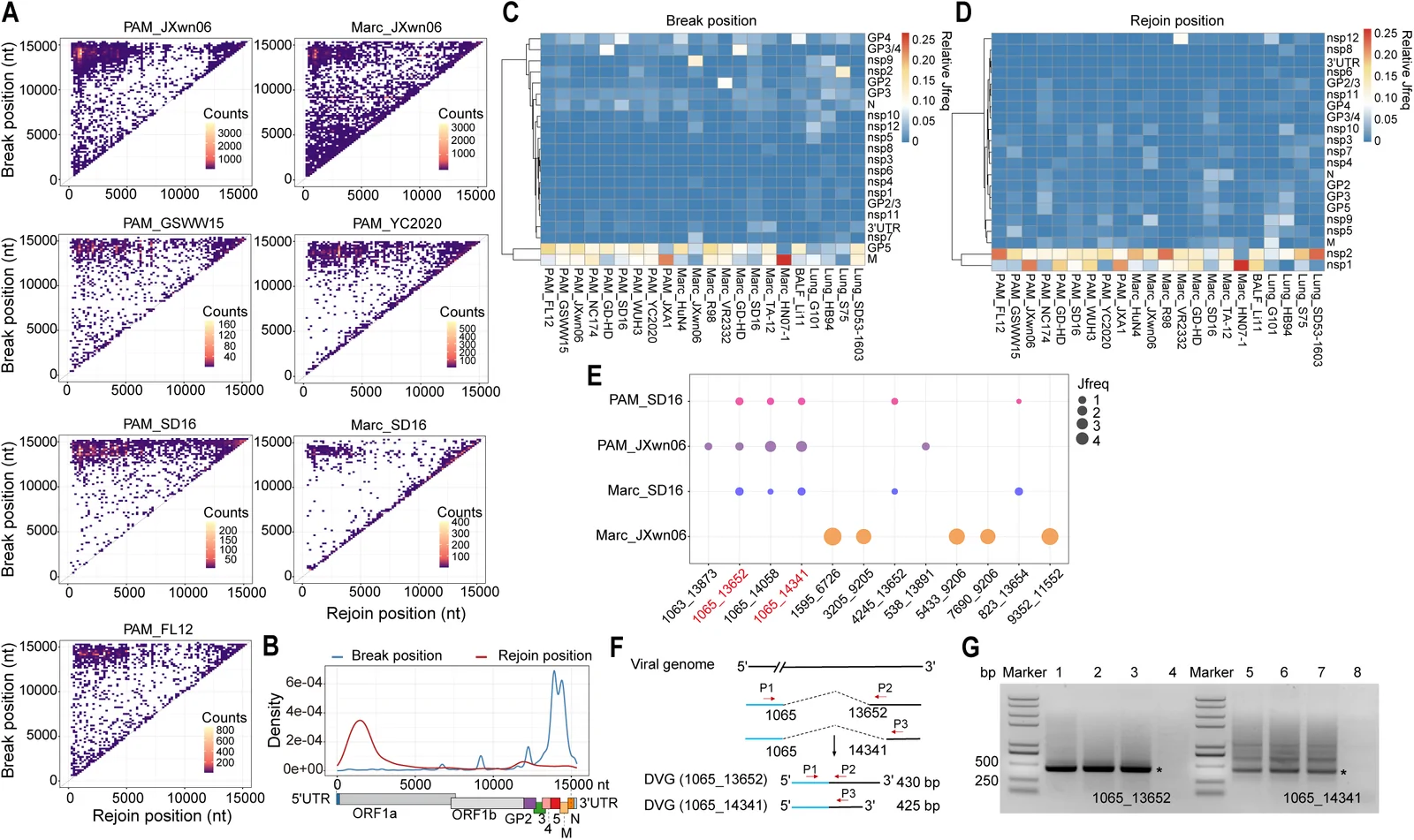
Analysis of DVG break and rejoin positions in PRRSV-2. **(A)** Distribution of DVG break and rejoin positions generated by different PRRSV-2 strains in infected cells. Each dot represents an individual DVG, plotted by its break position (y-axis) and rejoin position (x-axis), with color intensity indicating its relative abundance. **(B)** Analysis of the density of DVG break and rejoin positions weighted by Jfreq across different genomic regions. **(C–D)** Heatmaps showing the frequency distribution of DVG break positions **(C)** and rejoin positions **(D)** across different genomic regions in different viral strains. Strain names are indicated on the below, and the gene names are indicated on the right. Color intensity represents the relative Jfreq of DVG break and rejoin sites across different genomic regions within each strain. Relative Jfreq was calculated as the Jfreq of each DVG junction divided by the total Jfreq of all detected DVG junctions within the corresponding strain. The heatmap was visualized using log_10_ (Relative Jfreq + 1)-transformed values to reduce the influence of highly abundant DVG junctions. **(E)** The top five most abundant DVG species derived from the SD16 and JXwn06 strains in PAMs and Marc-145 cells. DVGs detected in more than three independent samples are shown in red. **(F)** PCR-based strategy for DVG detection. Primer pairs P1/P2 and P1/P3 were designed to specifically amplify DVG products with deletion ranges from 1,065 to 13,652 and from 1,065 to 14,341, respectively. **(G)** DVG-specific RT-PCR analysis of Marc-145 cells infected with the HuN4 strain (MOI = 0.1). The expected amplicon bands are indicated by black asterisks (*). Lanes 1–4: RT-PCR with DVG 1065_13652-specific primers using input virus stock (lane 1), infected-cell supernatant (lane 2), infected-cell lysate (lane 3), and uninfected cells (lane 4). Lanes 5–8: RT-PCR with DVG 1065_14341-specific primers using input virus stock (lane 5), infected-cell supernatant (lane 6), infected-cell lysate (lane 7), and uninfected cells (lane 8).

To further characterize the distribution of DVG break and rejoin positions, we performed subsequent analyses using junction frequency (Jfreq) to minimize the influence of differences in DVG abundance among samples and highly abundant DVG events. Jfreq was defined as the number of reads supporting a specific DVG junction per 10,000 viral reads in the corresponding sample. When combining all samples for density analysis of break and rejoin positions, the overall trend was consistent with single-strain analyses: break positions were mainly located within the genomic region encoding GP4 through N, while rejoin positions were concentrated in the left region of ORF1a (Fig 3B). To further quantify the distribution of DVG break and rejoin sites across viral genomic regions, we calculated the proportion of break and rejoin sites within each genomic region for each strain. The resulting heatmaps revealed that break positions were predominantly distributed in the GP5- and M-encoding regions, whereas rejoin positions were concentrated in the nsp1 and nsp2 regions (Fig 3C and 3D). These regional patterns complemented the spatial distribution and overall density of DVG junctions along the genome shown in Fig 3A and 3B, providing a quantitative assessment of DVG break and rejoin site preferences across defined viral genomic regions. Together, these analyses demonstrate that DVG break and rejoin sites exhibit reproducible regional preferences across PRRSV-2 strains.

To experimentally validate the identified DVGs *in vitro*, we selected four samples representing two HP-PRRSV lineage 8.7 strains (SD16 and JXwn06) in two cell types (Marc-145 cells and PAMs). The virus strains used in these samples are genetically similar to the HP-PRRSV lineage 8.7 HuN4 strain used for subsequent *in vitro* validation and exhibited relatively abundant and diverse DVGs. The five most abundant DVGs from each sample were then selected for further analysis. We found two DVGs, 1065_13652 and 1065_14341, that were consistently detected in three samples, with their break and rejoin positions located in high-frequency recombination regions (Fig 3E). Based on these recurrent junction sites, DVG-specific primers were designed, including P1 (840F), P2 (13855R), and P3 (14541R) (Fig 3F). RT-PCR analysis detected both the 1065_13652 and 1065_14341 DVGs in the input virus stock, as well as in culture supernatants and cell lysates collected from HuN4-infected Marc-145 cells (Fig 3G). No corresponding amplicons were detected in uninfected control cells. Sanger sequencing of the RT-PCR products confirmed the expected deletion junctions (S4 Fig). The 1065_13652 DVG was experimentally detected at 1065_13651, 1 nt shorter than the ViReMa-predicted junction at 1065_13652, whereas the second DVG was confirmed at 1065_14341.

### Large and small deletion DVGs exhibit distinct preferences for junctional microhomology

To determine whether the formation of PRRSV-2 DVGs is mediated by microhomology sequences like other RNA viruses [50,51], we examined whether the most frequent DVG break and rejoin positions in some samples exhibited sequence homology. With the exception of the Marc_SD16 sample, the most abundant DVG species in all other samples displayed clear microhomology, ranging from 4 to 12 nt in length (Fig 4A). These findings suggest that the formation of DVGs may be linked to microhomology-mediated recombination. To further investigate the role of microhomology in PRRSV-2 DVG formation, we first analyzed the sequence homology between break and rejoin sites of DVGs with different deletion lengths and visualized their distribution using heatmaps. For large deletion DVGs, break and rejoin sites in most samples were predominantly enriched in regions containing 4–6 nt of microhomology (Fig 4B). In contrast, small deletion DVGs exhibited a more heterogeneous distribution of microhomology lengths (Fig 4C). In several samples, including PAM_YC2020, Marc_JXwn06, Marc_TA-12, Marc_VR2332, and Lung_G101, the junctions of small deletion DVGs were mainly associated with 4–6 nt microhomology, whereas in the remaining samples they were more frequently distributed within the 0–3 nt range. To compare microhomology preferences between DVGs with different deletion lengths, we analyzed the cumulative distribution function (CDF) of microhomology length in large and small deletion DVGs. The CDF illustrates the distribution of microhomology lengths, where a rightward shift of the curve indicates a relative increase in longer microhomology sequences. In our analysis, large deletion DVGs showed a consistent rightward shift compared with small deletion DVGs, particularly across the 0–6 nt range (Fig 4D). At a given microhomology length, the cumulative proportion was consistently lower for large deletion DVGs, indicating that a smaller proportion of junction abundance in large deletion DVGs was accounted for by short microhomology sequences and that the distribution was shifted toward longer microhomology lengths. These results indicate that DVGs with large deletions were associated with relatively longer microhomology sequences at their junctions.

**Fig 4:**
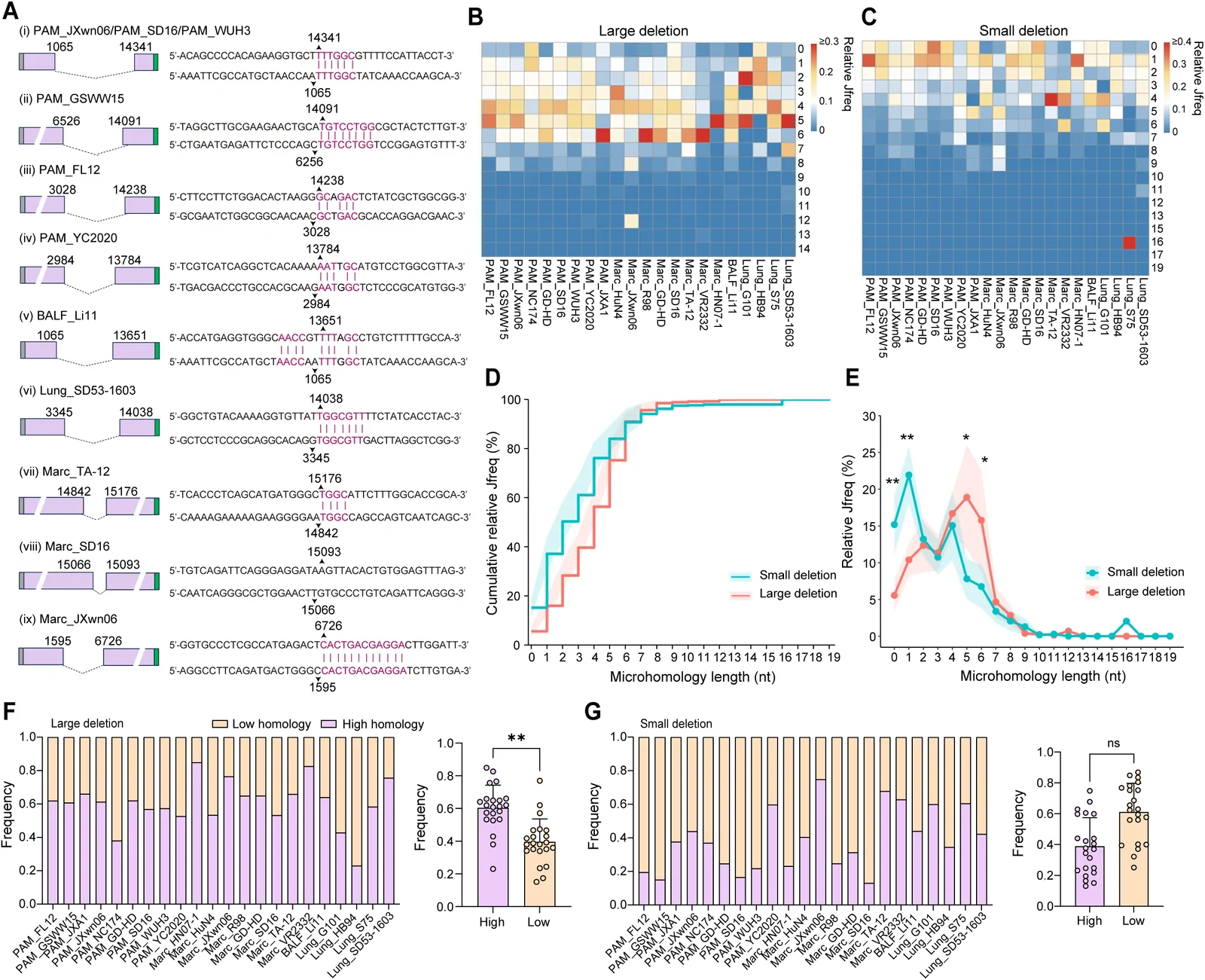
Microhomology sequence analysis. **(A)** Analysis of sequence homology surrounding the break and rejoin sites in highly abundant DVGs. The schematic on the left illustrates the relatively abundant DVG identified in each sample. The deleted internal region is indicated by a dashed line, with the retained 5′ and 3′ UTR shown on the left and right sides, respectively. The right panels display the nucleotide sequences flanking the break and rejoin sites. Sequences correspond to the positive-sense genomic strand of the respective viral strain and are shown in the 5′ to 3′ orientation. The nucleotides marked by a black arrow indicate the precise positions of break and rejoin. The same nucleotides located near the break and rejoin sites are highlighted in purple, and are connected by a vertical purple line. **(B–C)** Heatmaps showing the distribution of microhomology lengths at the break and rejoin positions of large deletion DVGs **(B)**, and small deletion DVGs **(C)**, across different PRRSV-2 strains. Strain names are indicated on the below, and the number of micro-homologous bases is indicated on the right. Color intensity represents the relative Jfreq associated with different microhomology lengths within each strain. **(D)** Cumulative distribution of junctional microhomology lengths in large deletion and small deletion DVGs. Curves represent the cumulative relative Jfreq across samples, and shaded areas indicate the 95% confidence intervals. **(E)** Relative abundance of large and small deletion DVGs across different microhomology lengths. Points and lines represent the mean relative Jfreq at each microhomology length across samples. Error bars indicate the 95% confidence intervals. Statistical significance was determined using paired Wilcoxon signed-rank tests, with *P* values adjusted for multiple comparisons using the Benjamini–Hochberg method. *, *P* < 0.05; **, *P* < 0.01. **(F–G)** The proportions of high- and low-microhomology events at the rejoin sites of large deletion DVGs **(F)**, and small deletion DVGs (G) were quantified and compared across strains. The overall microhomology among all strains is represented using a bar chart. Microhomology lengths ≥4 nt were defined as high microhomology, and <4 nt as low microhomology. Error bars represent the mean ± SD. Statistical significance was assessed using a two-tailed paired Student’s t-test. ns, not significant; **, *P* < 0.01.

We next compared the overall distribution of microhomology lengths between DVGs with different deletion sizes. The Jfreq-based distribution showed that small deletion DVGs had higher relative junction abundance at 0–1 nt microhomology lengths, whereas large deletion DVGs had higher relative junction abundance at 5–6 nt microhomology lengths (Fig 4E). Furthermore, both large deletion DVGs and small deletion DVGs contained junctions lacking detectable microhomology (microhomology length = 0), indicating that detectable microhomology was not present at all DVG junctions. To further evaluate this pattern, DVGs were classified into a high-microhomology group (microhomology length ≥4 nt) and a low-microhomology group (microhomology length <4 nt), and the relative proportions of the two groups were compared across samples from different lineages. For large deletion DVGs, the overall proportion of the high-microhomology group was significantly greater than that of the low-microhomology group (Fig 4F), further supporting a stronger association between microhomology and the formation of large deletion DVGs. In contrast, for small deletion DVGs, there was no significant difference between the proportions of the low- and high-microhomology groups (Fig 4G). Taken together, these results indicate that PRRSV-2 DVG formation does not strictly depend on microhomology, as DVGs can also be generated in the absence of detectable homologous sequences. Nevertheless, large deletion DVGs exhibited a stronger association with microhomology, whereas small deletion DVGs, although also containing microhomology-mediated junctions, were predominantly distributed within low-microhomology regions.

### RNA secondary structures contribute to the formation of PRRSV-2 DVGs with deletion ≤100 nt

We observed that the relationship between DVGs and microhomology was relatively complex, suggesting that factors other than microhomology may contribute to the formation of these DVGs. Previous studies have reported that RNA secondary structures may influence DVG generation during viral replication [52,53]. We therefore investigated whether RNA secondary structures are associated with the formation of DVGs in PRRSV-2. Because RNA secondary structure prediction algorithms such as RNAfold are more reliable for relatively short RNA sequences [54], we restricted the analysis to DVGs with deletion lengths ≤100 nt. DVGs with deletions <25 nt had been excluded during the initial filtering process to minimize the inclusion of small deletion events potentially generated by transient polymerase slippage. To quantify the relative abundance of individual DVG junctions within the viral population, we summed the Jfreq values of all DVG junctions with deletion lengths between 25 and 100 nt for each sample. Among the analyzed samples, Marc_SD16 exhibited the highest cumulative Jfreq (Fig 5A).

**Fig 5:**
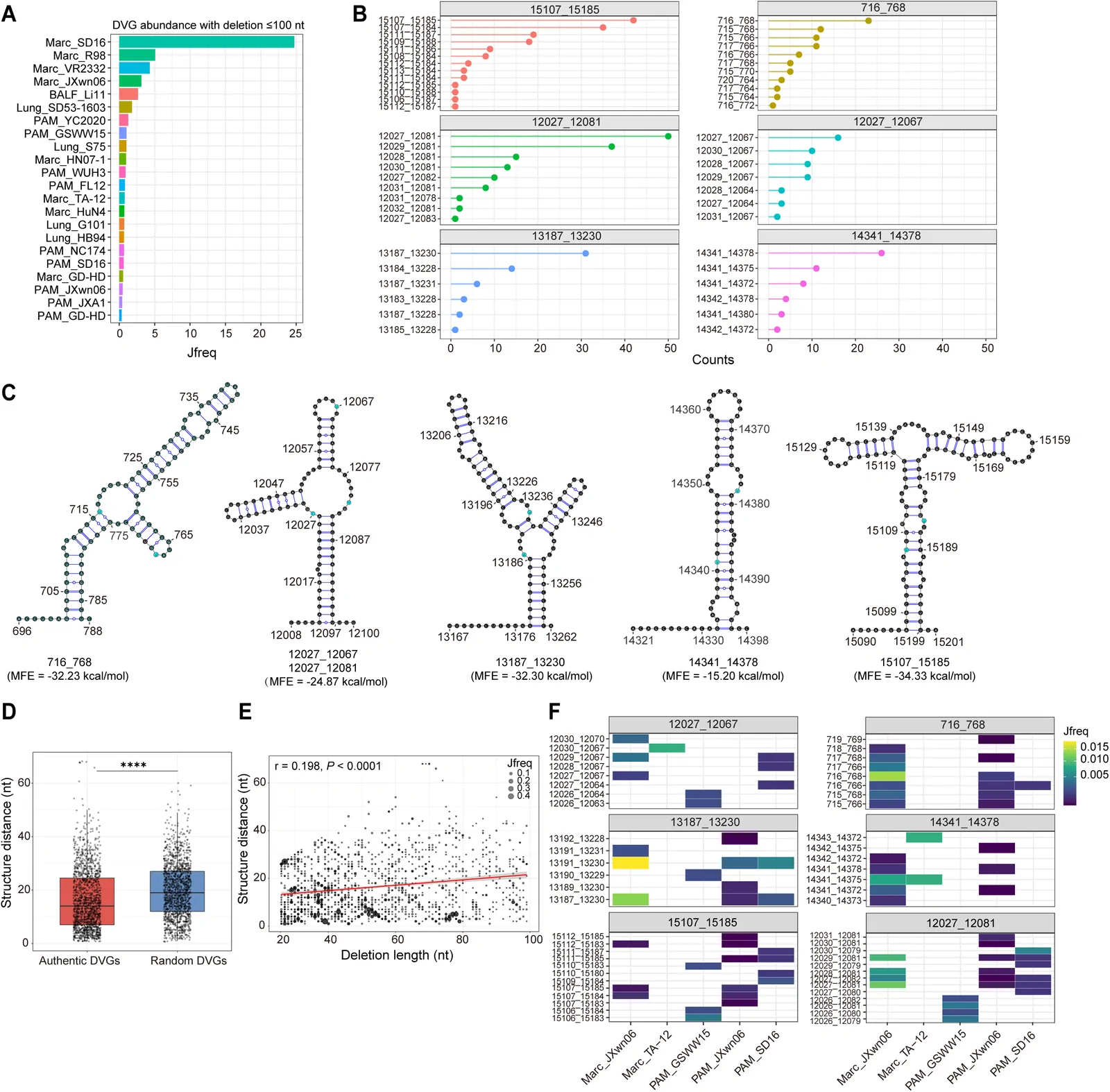
RNA secondary structure analysis of DVGs with deletion length ≤100 nt. **(A)** Abundance of DVGs with deletion lengths ≤100 nt across all samples. DVG abundance is represented by Jfreq. **(B)** Identification of recurrent high-frequency DVG junction hotspots in the Marc_SD16 sample. **(C)** Predicted RNA secondary structures surrounding the representative high-frequency DVG hotspots. The nucleotide positions shown in each structure correspond to the genomic coordinates of the PRRSV-2 SD16 genome. The values shown below each structure represent the MFE of the predicted structure, expressed in kcal/mol. **(D)** Comparison of RNA structural distances between break and rejoin points of authentic DVGs and randomly simulated DVGs in the Marc_SD16 sample. Statistical significance was assessed using a two-sided Wilcoxon rank-sum test. ****, *P* < 0.0001. **(E)** Pearson’s correlation analysis was used to assess the relationship between deletion length and RNA structural distance. The values *r* and *P* indicate Pearson’s correlation coefficient and the corresponding *P*-value, respectively. **(F)** Abundance of recurrent DVGs identified in the Marc_SD16 sample across samples from other lineage 8 strains.

We therefore selected the Marc_SD16 sample for subsequent analyses. Examination of recurrent DVG junctions identified six major hotspot regions, represented by junctions 15107_15185, 716_768, 12027_12081, 12027_12067, 13187_13230, and 14341_14378. Individual DVGs within each hotspot group differed by only 1–8 nt, indicating highly clustered junction distributions (Fig 5B). RNA secondary structures surrounding these hotspot regions were then predicted using RNAfold. All six hotspots were located within structurally complex RNA regions. Although the corresponding deletion lengths ranged from 37 to 78 nt, the RNA structural distances between break and rejoin points were only 5–11 nt, substantially shorter than their linear sequence distances (Fig 5C). These observations suggest that DVG break and rejoin points preferentially occur at positions that are spatially proximal within the folded RNA structure. To further evaluate this association, we compared the structural distances of authentic DVG junctions with those of randomly simulated DVGs having the same deletion lengths. Authentic DVGs exhibited significantly shorter RNA structural distances than the random controls (Fig 5D), indicating that naturally occurring DVG junctions preferentially connect RNA regions that are close in secondary structure rather than being randomly distributed along the viral genome. We next examined the relationship between deletion length and RNA structural distance. Shorter deletions tended to occur between RNA regions with shorter structural distances, although the correlation was relatively weak (r = 0.198, *P* < 0.0001) (Fig 5E). Finally, we investigated whether these recurrent DVG junctions were conserved among other lineage 8 strains. These recurrent DVG junctions were initially identified in the Marc_SD16 sample, and the SD16 strain belongs to lineage 8. Therefore, strains within the same lineage provide a more appropriate genetic context for assessing the conservation of these recurrent DVG junctions. Similar DVG junctions were detected in several additional samples derived from lineage 8 strains, including Marc_JXwn06, Marc_TA-12, PAM_GSWW15, PAM_JXwn06, and PAM_SD16, supporting the conservation of these recurrent DVG junctions among lineage 8 strains (Fig 5F). These findings indicate that the identified hotspots were repeatedly detected across multiple lineage 8 strains rather than representing random recombination events, suggesting that common mechanisms may contribute to hotspot formation.

### DVG generation is associated with AU-rich sequences

Beyond microhomology sequences and RNA secondary structures, DVG breakpoints in several RNA viruses have been reported to preferentially occur in AU-rich regions [29,51,55]. We calculated the AU content of the 20 nucleotides flanking DVG break and rejoin sites for each PRRSV-2 strain. Twelve out of 22 PRRSV-2 strains from cell and tissue samples displayed a notable enrichment of AU content and a corresponding depletion of GC content within −5 to +5 nt around the break and rejoin positions (Fig 6). Overall, break and rejoin sites in these strains are enriched in AU-rich regions, indicating that PRRSV-2 DVG formation, like other RNA viruses, is closely associated with AU-rich sequences.

**Fig 6:**
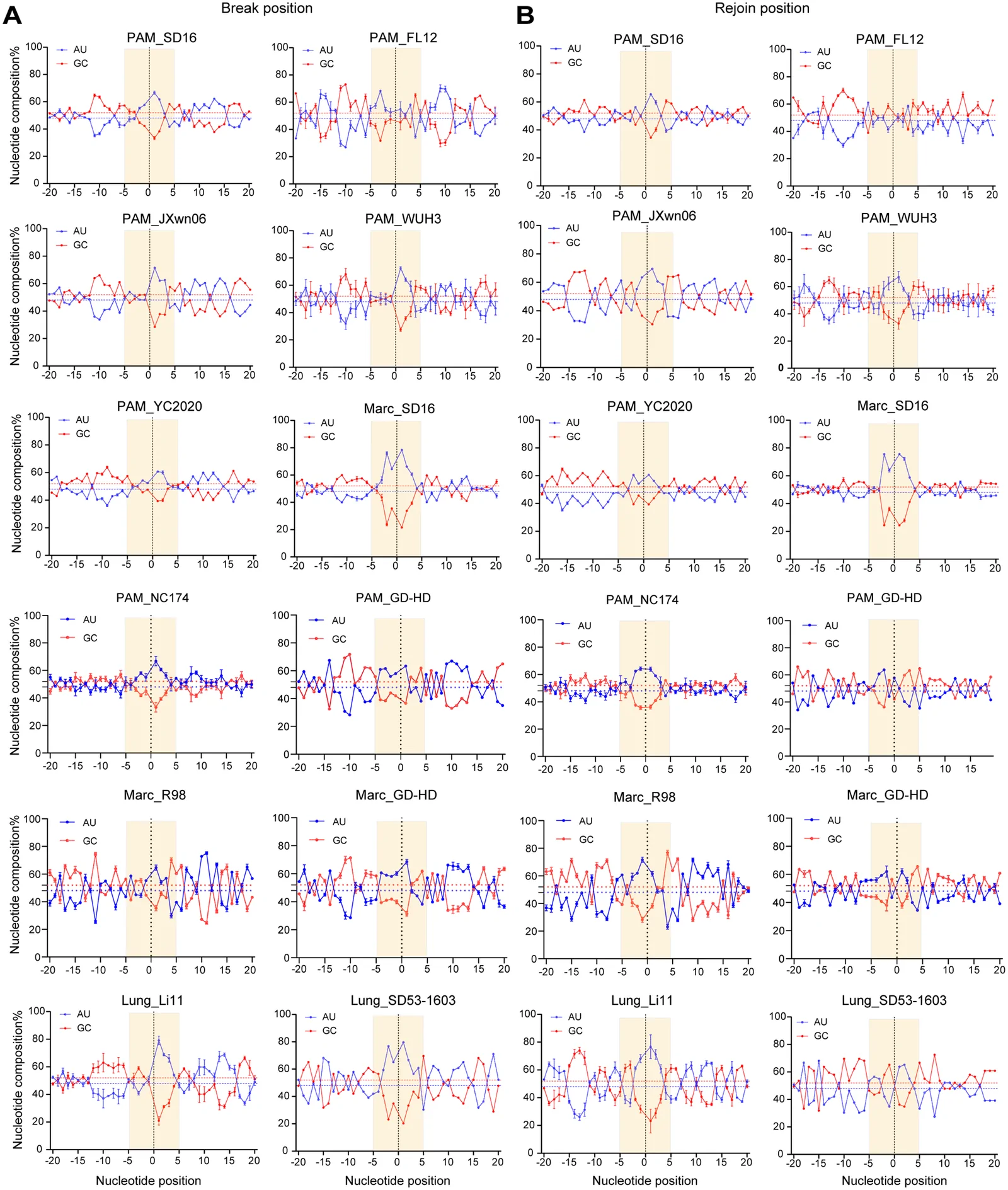
Analysis of nucleotide composition at break and rejoin positions. **(A–B)** Nucleotide composition surrounding DVG break positions **(A)** and rejoin positions **(B)** was analyzed by calculating the percentages of AU and GC at each position within a 20-nt window flanking the respective position. Positions upstream of the junction were designated −20 to −1, and downstream positions were labeled +1 to +20. AU and GC contents at each position were calculated from all DVG break positions or rejoin positions within each sample. The AU content at each position is shown as a solid red line, and the GC content is shown as a solid blue line. The expected AU and GC frequencies based on the complete viral genome sequence are indicated by red and blue dashed lines, respectively. The shaded region indicates the −5 to +5 nt window surrounding the DVG junctions. Data are presented as mean ± SD.

### Naturally occurring PRRSV-2 nsp2 deletion variants are associated with DVG formation

Our preceding analyses showed that DVG junctions were non-randomly distributed across the PRRSV-2 genome and exhibited distinct sequence and structural features, including differences in microhomology, RNA secondary structure, and local nucleotide composition. Because DVGs represent deletion-containing forms of the viral genome, we next asked whether the genomic regions associated with DVG deletions also corresponded to regions in which deletion variants occur naturally or emerge during virus passage. In particular, DVG junctions were frequently located within the nsp2 region, prompting us to further examine deletion patterns within this gene (Fig 3D). PRRSV-2 nsp2 is characterized by high genetic variability and contains a prominent hypervariable region in its central domain. This region frequently contains deletion mutations that are observed in multiple PRRSV-2 variants, including NADC30-like, NADC34-like, and HP-PRRSV strains [10,11]. The frequent occurrence of both DVG junctions and deletion variants within nsp2 suggests that this genomic region may be particularly permissive to deletion events. To further characterize this pattern, we first analyzed the frequency of deletion events occurring within the same nonstructural protein gene among PRRSV-2 strains from different lineages. The results showed that nsp2 contributed a substantial proportion of intragenic deletion events (Fig 7A). We next mapped the spatial distribution of DVG junctions within the nsp2 region. DVGs identified in lineage 1, 5, and 8 strains were predominantly clustered along the diagonal, indicating that breakpoints and rejoin points tended to occur within relatively nearby genomic regions (Fig 7B). Among these lineages, lineage 8 strains exhibited a greater diversity of DVG species, which may be attributable to the larger number of lineage 8 strains included in this study.

**Fig 7:**
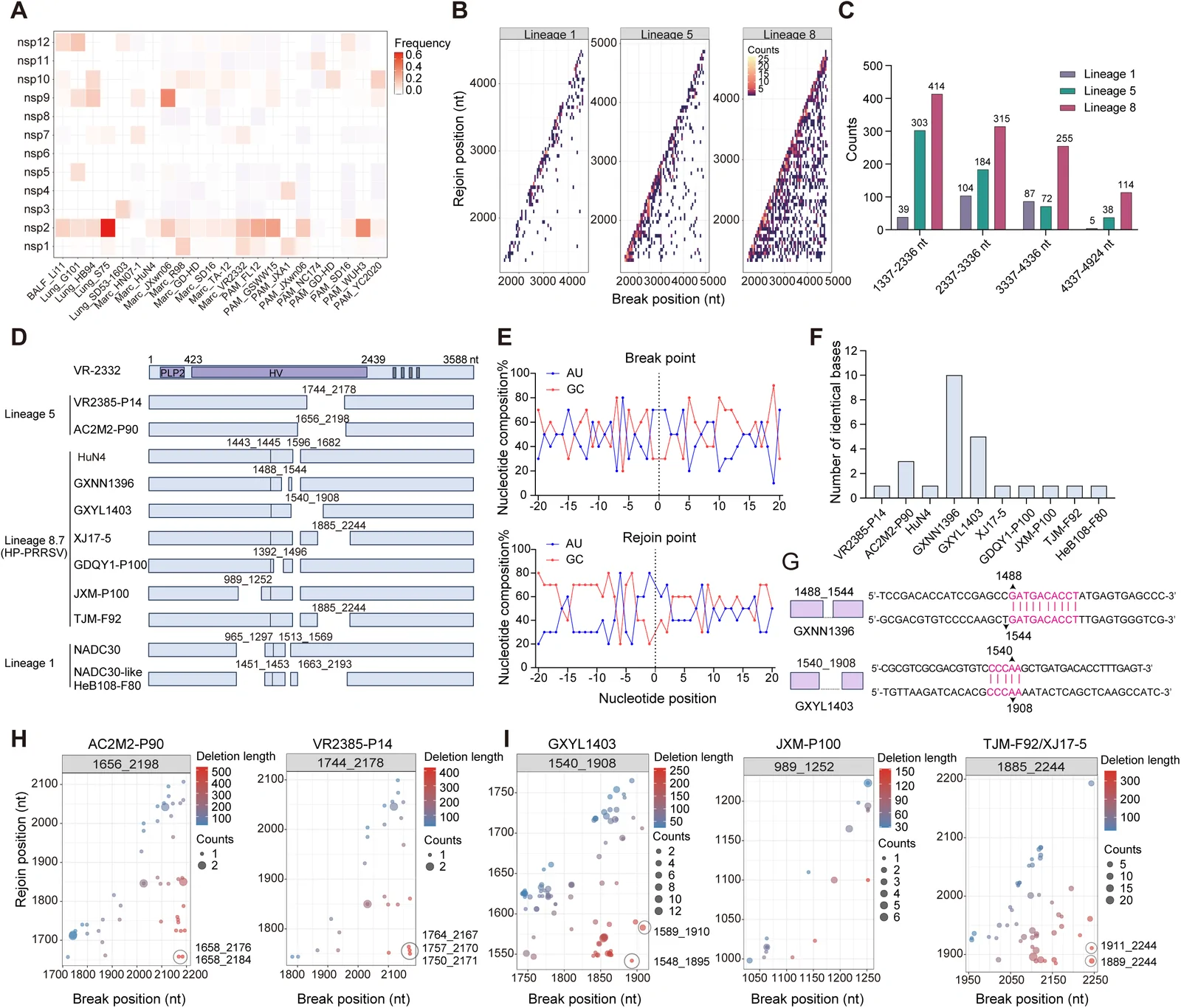
Natural PRRSV-2 nsp2 deletion genomes exhibit DVG-like features. **(A)** Heatmap showing the frequency of deletion events occurring within the same nonstructural protein gene among different PRRSV-2 strains. The analyzed strains included thirteen lineage 8 strains, six lineage 1 strains, and five lineage 5 strains. **(B)** Distribution of DVGs within the nsp2 region in PRRSV-2 strains belonging to lineages 1, 5, and 8. **(C)** The nsp2 region was divided into four segments of 1000 nt each, and the number of DVGs distributed within each segment was quantified. **(D)** Schematic representation of nsp2 deletion positions in PRRSV-2 and passaged PRRSV-2 strains from different lineages. The full-length nsp2 region was defined according to the reference strain VR-2332. Break and rejoin positions are connected by “_”. Black vertical lines within nsp2 indicate single-amino-acid deletions. **(E)** Analysis of AU content surrounding nsp2 deletion regions in WT virus and passaged PRRSV-2 strains from different lineages. **(F)** Analysis of microhomologous sequences flanking nsp2 deletion sites in strains with corresponding parental viruses. **(G)** Schematic diagram of homologous sequences at the deletion sites of GXNN1396 and GXYL1403. The nucleotides marked by a black arrow indicate the precise positions of break and rejoin. The same nucleotides located near the break and rejoin sites are highlighted in rose red, and are connected by a vertical red line. **(H** and **I)** Distribution map showing the correspondence between RNA-seq-derived DVGs and naturally occurring nsp2 deletion regions. The figure titles indicate the genomic positions of the nsp2 deletion ranges shown in panel **(D)**. DVGs with similar deletion ranges are highlighted by circles.

To determine which regions of nsp2 were more prone to deletion, the nsp2 region of the VR-2332 reference strain was divided into four segments: 1337–2336 nt, 2337–3336 nt, 3337–4336 nt, and 4337–4924 nt. The number of DVGs identified within each segment was then quantified. DVGs were predominantly detected within the first 2000 nt of nsp2, particularly within the first 1000 nt (Fig 7C). To further investigate the relationship between naturally occurring nsp2 deletion variants and DVGs, we mapped a schematic of representative nsp2 deletion strains (Fig 7D). Two lineage 5 cell-passaged strains, VR2385-P14 and AC2M2-P90, contained deletions exceeding 400 nt. The characteristic nsp2 deletion patterns of lineage 8 and lineage 1 PRRSV-2 strains are also shown, including the 1-aa and 29-aa deletions in HP-PRRSV (1443–1445 nt and 1596–1682 nt, respectively) and the 111-aa, 1-aa, and 19-aa deletions in NADC30-like strains (965–1297 nt, 1451–1453 nt, and 1513–1569 nt, respectively). These characteristic deletions have been used as molecular markers to distinguish these viral variants [15,56]. In addition to these characteristic deletions, additional deletions at distinct genomic locations were identified from available sequences of lineage 8 and lineage 1 strains, including the field isolates GXNN1396, GXYL1403, and XJ17–5, as well as the cell-passaged strains GDQY1-P100, JXM-P100, TJM-F92, and HeB108-F80 (Fig 7D). We subsequently extracted 40-nt sequences flanking the break and rejoin sites of these naturally occurring nsp2 deletions for AU-content and microhomology analyses. We found that the sequences surrounding the deletion regions exhibited high AU content (Fig 7E). In addition, GXNN1396 and GXYL1403 strains contained 10-nt and 5-nt homologous sequences, respectively, between their break and rejoin sites (Fig 7F and 7G), indicating that naturally occurring nsp2 deletions share sequence characteristics commonly associated with DVG formation.

To further examine whether the naturally occurring nsp2 deletion regions corresponded to the deletion regions represented by the identified DVGs, we mapped the DVG deletion regions onto the nsp2 region and compared their spatial correspondence (Fig 7H and 7I). In lineage 5 strains, the region spanning 1656_2198 nt (corresponding to the AC2M2-P90) contained multiple DVGs, including two unique DVG events (1658_2176 and 1658_2184 nt) with deletion boundaries highly similar to the naturally occurring nsp2 deletion. Likewise, within the 1744–2178 nt region (corresponding to the VR2385-P14), three related DVG events (1764_2167, 1757_2170, and 1750_2171 nt) were identified. In lineage 8 strains, within the 1540–1908 nt region (corresponding to the GXYL1403), two related DVG events (1589_1910 and 1548_1895 nt) were identified. Interestingly, although no DVG with an identical deletion size was identified within the 989–1252 nt region (corresponding to the JXM-P100), multiple DVGs shared the same rejoin site at 1252 nt, suggesting that this region may represent a hotspot for template switching. Notably, in the 1885–2244 nt region (corresponding to the TJM-F92/XJ17–5), two additional related DVG events (1911_2244 and 1889_2244 nt) were detected, among which the rejoin site of DVG 1889_2244 differed from the naturally occurring nsp2 deletion boundary by only four nucleotides. Taken together, these findings demonstrate that naturally occurring nsp2 deletion regions in PRRSV-2 not only exhibit sequence characteristics similar to those associated with DVG formation, but also overlap with DVG junction events identified by RNA-seq, suggesting that some nsp2 deletion variants may arise through DVG-like recombination mechanisms.

### DVG interferes with the replication of PRRSV-2 *in vitro*

To evaluate the antiviral activity of DVGs generated during PRRSV-2 infection, we constructed plasmids expressing different DVG sequences and assessed their impact on viral replication in Marc-145 cells. Previous studies have indicated that high-abundance and repeated DVGs across multiple sequencing datasets are more likely to possess antiviral activity [57]. In Fig 3, we observed higher frequencies of the 1065_13652 and 1065_14341 DVGs in HP-PRRSV strains, both detectable in HuN4-infected cells using RT-PCR. Therefore, these two high-abundance DVGs (DVG1–2) with distinct deletion patterns were selected for antiviral analysis (Fig 8A). To exclude the possibility that the plasmids expressing partial coding sequences themselves might impair viral replication, a control plasmid encoding a partially truncated but frameshifted ORF3–6 sequence derived from PRRSV-1 was also constructed. Before evaluating antiviral activity, we first examined the expression of these plasmids in Marc-145 cells. The results showed that DVG1, DVG2, and the control plasmid were expressed in transfected Marc-145 cells (Fig 8B). To determine whether DVGs exert antiviral effects, Marc-145 cells were transfected with DVG plasmids, control plasmid, or empty vector and subsequently infected with PRRSV-2 HuN4 at an MOI of 0.1. Viral titers were quantified at 48 h post-infection (hpi). All DVGs significantly reduced viral titers, whereas neither the control plasmid nor the empty vector showed an inhibitory effect on viral replication (Fig 8C). To determine whether DVG1 retains antiviral activity post-entry, cells were first infected with PRRSV-2 HuN4 strain (MOI = 0.1) and transfected with DVG1 at 8 hpi. Viral RNA levels and titers were assessed at 48 hpi. Even when delivered after viral infection, DVG1 significantly reduced viral replication (Fig 8D), indicating that it can inhibit viral replication during the later stages of infection.

**Fig 8:**
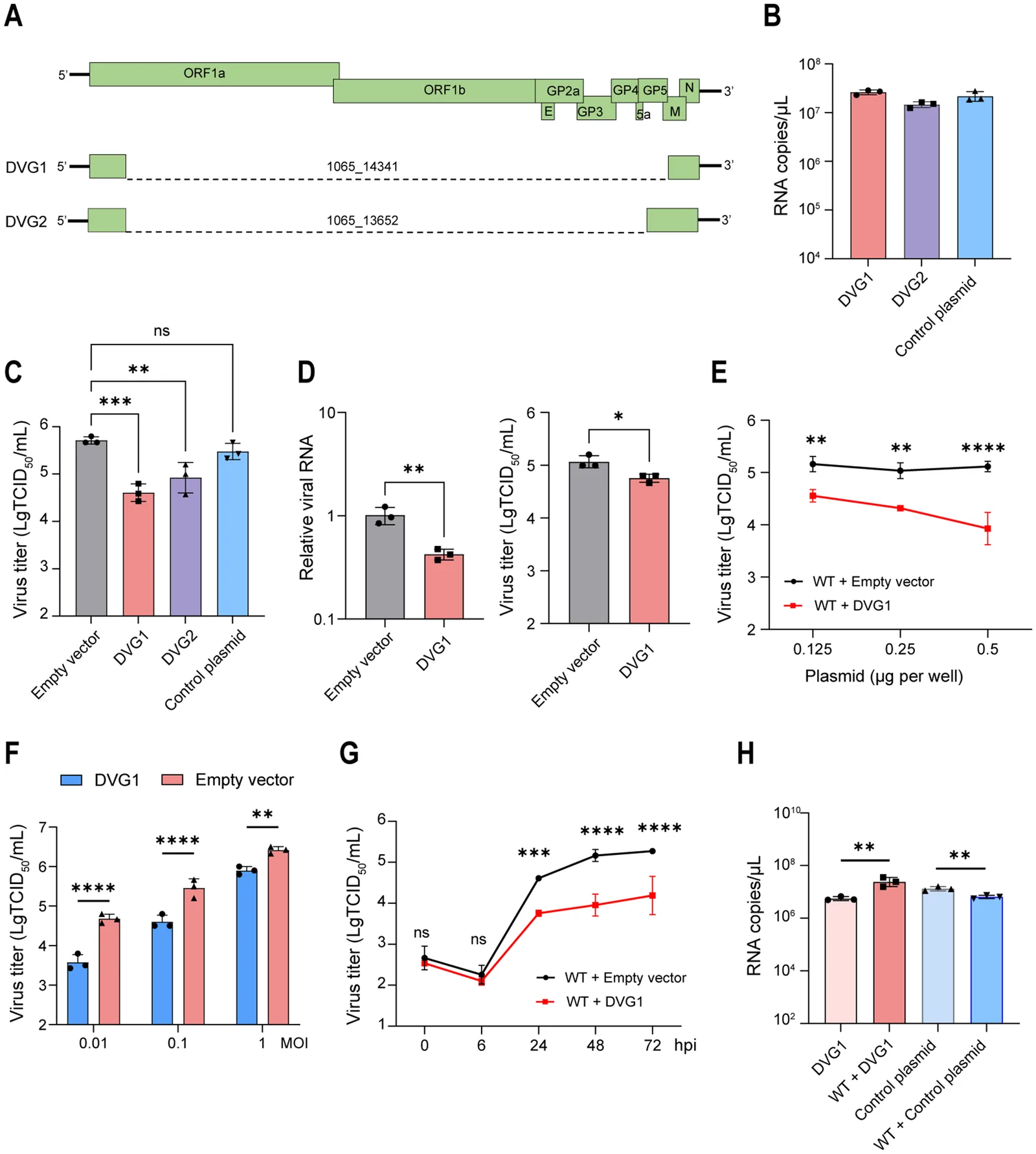
DVGs interfere with the replication of PRRSV-2 *in vitro.* **(A)** Schematic representation of the engineered DVG1 to DVG2 constructs. Dashed lines indicate deleted regions. For each DVG, the numbers flanking the “_” denote the rejoin and break sites, respectively. All engineered DVGs retain the authentic 5′ and 3′ UTR sequences of PRRSV-2. **(B)** DVG mRNA expression in Marc-145 cells. Marc-145 cells were transfected with DVG1, DVG2, or control plasmids. At 24 hpt, RNA levels were measured by RT-qPCR. **(C)** *In vitro* evaluation of the antiviral activity of DVGs. Marc-145 cells were transfected with 0.5 μg of DVG plasmid, control plasmid and empty vector, respectively. At 24 hpi, cells were infected with the PRRSV-2 HuN4 strain (MOI = 0.1), and viral titers were quantified at 48 hpi. **(D)** Antiviral efficacy of DVG1 in post-infection setting in cell culture. Marc-145 cells were infected with PRRSV-2 at MOI = 0.1 and transfected with DVG1 or empty vector at 8 hpi. Viral titers in supernatants were measured, and viral RNA levels were quantified at 48 hpi using RT-qPCR targeting the GP5 gene (absent in DVG1). Relative viral RNA was normalized to *GAPDH*. **(E)** Dose-dependent antiviral activity of DVG1. Marc-145 cells were transfected with increasing doses (0.125, 0.25, and 0.5 μg) of the DVG1 plasmid or empty vector, followed by infection with PRRSV-2 HuN4 strain (MOI = 0.1). Viral titers in supernatants were measured at 48 hpi. **(F)** Effect of DVG1 on PRRSV-2 replication at different MOIs. Marc-145 cells were transfected with 0.5 μg of empty vector or DVG1 plasmid and subsequently infected with PRRSV-2 HuN4 strain at MOIs of 0.01, 0.1, or 1. Viral titers were determined at 48 hpi. **(G)** Antiviral activity of DVG1 across different stages of the viral replication cycle. Marc-145 cells transfected with DVG1 were infected with PRRSV-2 (MOI = 0.1), and cell supernatants were collected at 0, 6, 24, 48, and 72 hpi for TCID_50_ titration. **(H)** DVG1 RNA accumulation in the presence or absence of WT PRRSV-2. Marc-145 cells were transfected with 0.5 μg of DVG1 or control plasmid and subsequently infected with PRRSV-2 or left uninfected. DVG1 RNA and control plasmid RNA levels were quantified by RT-qPCR at 24 hpi. For all panels, data represent mean ± SD from three independent experiments. C, H, one-way ANOVA with Dunnett’s test. D, unpaired t-test. E, F, G, two-way ANOVA with Sidak’s test. ns, not significant; *, *P* < 0.05; **, *P* < 0.01; ***, *P* < 0.001; ****, *P* < 0.0001.

To further investigate whether the inhibitory effect of DVG1 was dose-dependent, Marc-145 cells were transfected with increasing amounts of DVG1 plasmid and subsequently infected with PRRSV-2. DVG1 significantly suppressed viral replication at all tested doses, with greater inhibition observed at higher plasmid doses (Fig 8E). A significant treatment-by-dose interaction was detected (*P* = 0.0257), supporting a dose-dependent inhibitory effect of DVG1 on viral replication. To exclude the possibility that the antiviral activity of DVG1 was attributable to the low MOI used, Marc-145 cells were transfected with either empty vector or DVG1 plasmid, followed by PRRSV-2 infection at MOIs of 0.01, 0.1, or 1. DVG1 significantly inhibited PRRSV-2 replication at all three MOIs, although the inhibitory effect progressively decreased with increasing MOI (Fig 8F). To further characterize the temporal antiviral activity of DVG1, Marc-145 cells were transfected with either the empty vector or DVG1 plasmid and subsequently infected with PRRSV-2 at an MOI of 0.1. DVG1 significantly reduced viral titers at 24, 48, and 72 hpi compared with the empty vector control (Fig 8G).

To determine whether DVG1 can replicate in the presence of WT virus, we quantified DVG1 RNA levels in cells transfected with DVG1 plasmid in the presence or absence of WT PRRSV-2. Cells transfected with the control plasmid, with or without WT virus infection, were included in parallel. Compared with cells transfected with DVG1 alone, DVG1 RNA copy numbers were significantly increased in cells co-harboring DVG1 and WT virus, whereas the RNA copy numbers of the control plasmid were significantly decreased in the presence of WT virus (Fig 8H). These results showed that the presence of WT PRRSV-2 was associated with increased DVG1 RNA levels in infected cells.

### DVG exhibits broad-spectrum inhibition of PRRSV-2 replication across viral lineages

To determine whether this inhibitory effect extends to other PRRSV-2 lineages, we evaluated the antiviral activity of DVG1 against representative PRRSV-2 strains, including the HP-PRRSV HuN4 strain (lineage 8), the NADC30-like strain HeB108 (lineage 1C), and the NADC34-like strain HLJ29 (lineage 1A). Marc-145 cells were transfected with DVG1 or empty vector and subsequently infected with PRRSV-2. Quantification by RT-qPCR and TCID₅₀ assays showed that DVG1 significantly reduced viral RNA levels and viral titers in the HuN4, HeB108, and HLJ29 strains, indicating that DVG1 inhibits PRRSV-2 replication independent of lineage background (Fig 9A–9C). To further assess the breadth of this inhibitory activity, we extended our analysis to PRRSV-1, which belongs to a different species within the same genus. Using the PRRSV-1 DV strain, we observed a similar reduction in viral replication in DVG1-transfected cells, as measured by both viral RNA level and viral titer (Fig 9D). Together, these results demonstrate that DVG1 exhibits broad-spectrum antiviral activity, effectively suppressing viral replication across multiple-lineage PRRSV-2 and PRRSV-1.

**Fig 9:**
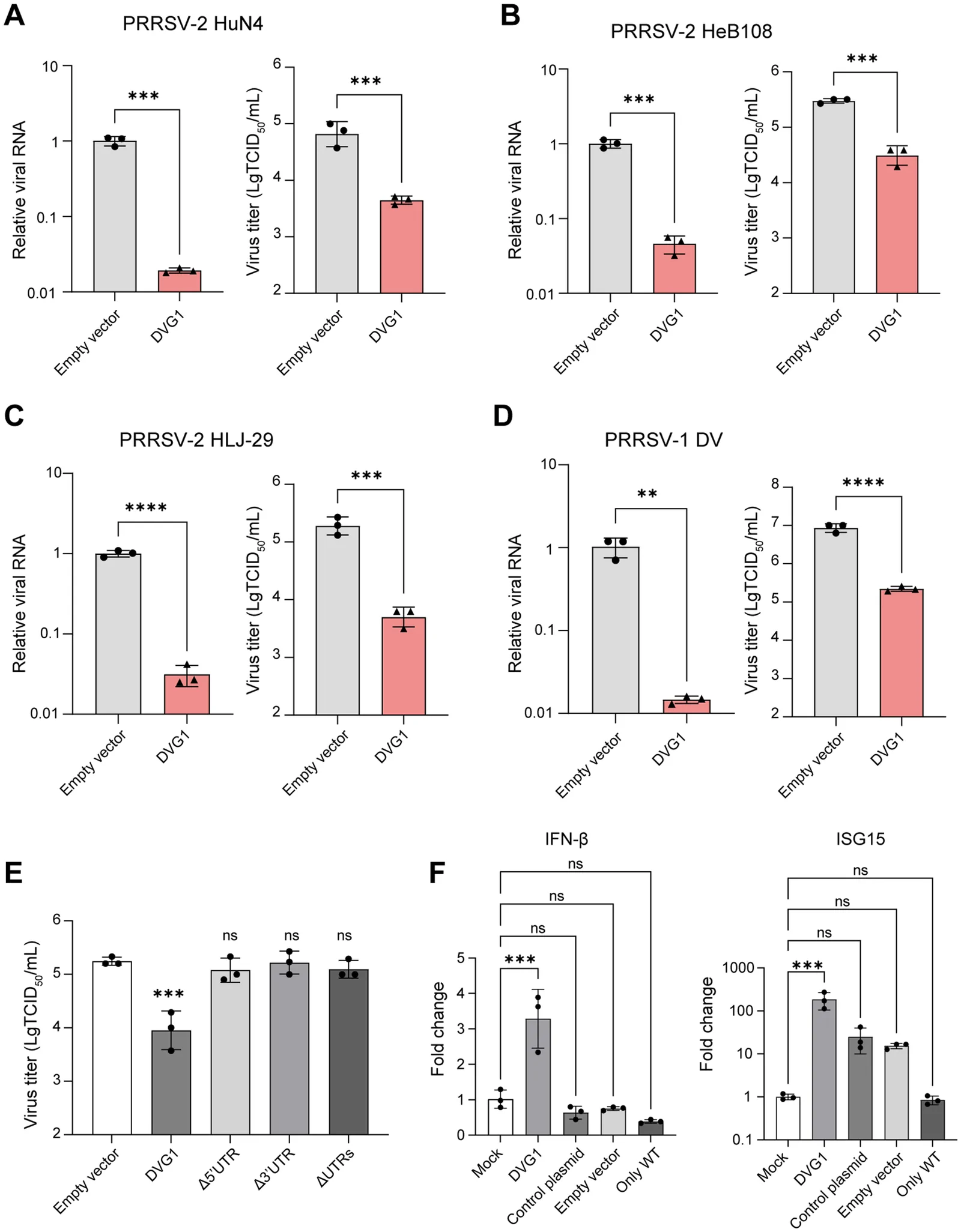
DVGs exhibit broad-spectrum inhibitory activity against PRRSV-2 replication. **(A–D)** *In vitro* evaluation of the antiviral activity of engineered DVG1 against HP-PRRSV strain HuN4 **(A)**, NADC30-like strain HeB108 **(B)**, NADC34-like strain HLJ-29 **(C)**, and PRRSV-1 strain DV **(D)**. Marc-145 cells were transfected with either DVG1 plasmid or empty vector and infected with PRRSV-2 (MOI = 0.1) at 24 hpt. At 48 hpi, viral titers in cell-culture supernatant were determined. Total RNA was extracted from the remaining cells, and was subjected to RT-qPCR targeting the GP5 gene to quantify viral RNA levels. **(E)** Antiviral activities of distinct DVG elements. Marc-145 cells were transfected with the full-length DVG1, DVG1 derivatives lacking the 5′ UTR (Δ5′UTR DVG1), 3′ UTR (Δ3′UTR DVG1), or both terminal untranslated regions (ΔUTRs DVG1), or empty vector, followed by PRRSV-2 infection. Viral titers in supernatants were measured at 48 hpi. **(F)** Detection of *IFN-β* and *ISG15* mRNA levels in PRRSV-2-infected Marc-145 cells. Marc-145 cells were transfected with either DVG1, control plasmid, or empty vector, followed by infection 24 h later with PRRSV-2 (MOI = 0.1). At 24 hpi, cells were harvested for the detection of antiviral gene expression. Data are presented as fold change relative to mock-treated cells. For all panels, data represent mean ± SD from three independent experiments. A–D, unpaired t-test. E–F, one-way ANOVA with Dunnett’s test. ns, not significant; **, *P* < 0.01; ***, *P* < 0.001; ****, *P* < 0.0001..

To determine whether complete DVG structures were required for antiviral activity, we generated a series of truncated DVG derivatives, including constructs lacking the 5′UTR (Δ5′UTR DVG), lacking the 3′UTR (Δ3′UTR DVG), or lacking both terminal untranslated regions (ΔUTRs DVG), and subsequently evaluated their antiviral effects. The results showed that only DVGs containing intact 5′ and 3′ UTRs were capable of suppressing viral replication (Fig 9E), indicating that loss of the terminal UTRs impaired the ability of DVGs to compete with WT virus for replication resources. DVGs from other viruses, particularly copy-back DVGs from negative-sense RNA viruses, have been reported to inhibit viral replication through activation of innate immune responses [31,58]. Therefore, we further examined the expression of two antiviral genes in cells co-harboring DVG1 and PRRSV-2. The expression levels of *IFN-β* and *ISG15* were significantly upregulated in DVG1-transfected cells, whereas no significant differences were observed in cells transfected with the control plasmid or empty vector (Fig 9F).

## Discussion

DVGs are accidental defective byproducts of RNA virus replication, but their ubiquity and diverse roles during infection indicate that they are an integral part of the viral life cycle. Studies across diverse RNA viruses have shown that DVGs are generated during both *in vitro* and *in vivo* infections and can influence viral replication and host innate immune responses [24,25]. Although several studies have reported the generation of DVGs during PRRSV-2 infection [44–46], our understanding of how these DVGs are formed and whether they exert biological functions remains limited. By integrating RNA-seq datasets derived from both *in vitro* and *in vivo* PRRSV-2 infection models, our study provides new insights into the formation characteristics of naturally occurring DVGs during PRRSV-2 infection. Together, our findings suggest that DVG generation during PRRSV-2 infection is influenced by host cell type and exhibits distinct break-rejoin characteristics, rather than arising solely from random replication errors.

The generation of DVGs during both *in vitro* and *in vivo* infections has been documented for a wide range of RNA viruses, including influenza virus [51,59], measles virus [60], respiratory syncytial virus [40,61], Ebola virus [62], and coronaviruses [29,55]. Our findings extend these observations to PRRSV-2, demonstrating that PRRSV-2 likewise produces DVGs in both *in vitro* and *in vivo*. Notably, cell culture samples yielded DVGs with higher abundance and diversity than tissue samples. It is known that serial passage of RNA viruses in cells at a high MOI can favor the generation and accumulation of DVGs within viral populations [26–28]. Therefore, differences in MOI among the datasets analyzed in this study may have contributed to variations in DVG abundance and diversity. However, because the RNA-seq datasets analyzed in this study differed in virus strain, infection time, and experimental system, the independent contribution of MOI to the observed differences in DVG abundance could not be assessed. Analysis of DVG deletion lengths revealed a distinct pattern, indicating that DVG formation is not random but exhibits specific deletion size preferences. In PAMs, DVGs more frequently exhibited large deletions (≥4000 nt), suggesting that PRRSV-2 in natural host-relevant cells may preferentially generate shorter DVG genomes. Previous studies have suggested that shorter DVG genomes may facilitate the maintenance of high replication efficiency, which may contribute to their higher abundance [27], consistent with our observations. Samples from lung tissue did not show this trend, possibly due to the more complex *in vivo* environment.

Microhomology-driven template switching has been shown to promote DVG formation in multiple RNA viruses, with break and rejoin sites often flanked by short homologous sequences that provide ‘anchors’ for the viral polymerase [50,51]. Our data indicate that PRRSV-2 DVGs also exhibit enrichment of repeated sequences, particularly in large deletion DVGs. Interestingly, small deletion DVGs displayed more heterogeneous homology patterns: some events were strongly microhomology-dependent, whereas others occurred with little to no homologous sequence—a phenomenon not systematically described in previous DVG studies. The relationship between RNA secondary structure and DVG formation appears to be complex. We found that, among DVGs with deletion lengths of ≤100 nt, break and rejoin sites preferentially occurred at positions that were spatially closer within the predicted RNA secondary structures, suggesting that local RNA structures may contribute to the selection of DVG junction sites. However, as this analysis was restricted to relatively short deletion DVGs and RNA secondary structure prediction has inherent limitations, these findings do not demonstrate that RNA secondary structure directly determines DVG formation. Overall, these results suggest that DVGs with different deletion lengths may exhibit distinct formation preferences and are likely influenced by multiple factors. Future studies will be required to elucidate the mechanisms underlying the generation of DVGs with different deletion lengths.

In addition, we note that DVG break and rejoin sites of some PRRSV-2 strains are enriched in AU sequences, consistent with previous reports in coronaviruses, RSV and influenza viruses [29,51,55,63]. Our study identified multiple high-frequency deletion hotspots in PRRSV-2 DVGs, indicating that their formation is regionally biased rather than random. Yuan et al. reported that PRRSV-2 DI RNAs could undergo deletions between the nsp1/nsp2 and nsp10 regions, but the precise hotspot locations were not systematically described [44–46]. Our results show that while DVGs can theoretically occur at any genomic position, rejoin positions are preferentially clustered within the genomic regions encoding nsp1 and nsp2, whereas break positions are primarily located in the genomic regions encoding GP5 and M. Notably, in our *in vitro* DVG amplification experiments, the DVGs 1065_13652 and 1065_14341 were detectable in the input HuN4 virus stock and were subsequently detected in both the culture supernatant and cell lysate of infected Marc-145 cells. Therefore, their detection during this infection does not demonstrate *de novo* generation, as they may have been carried over from the input virus stock and subsequently maintained or amplified during viral replication. Although these DVGs must have originated from *de novo* template switching events at an earlier stage, the DVG populations detected during subsequent infections may comprise both pre-existing and newly generated DVGs and may progressively accumulate through successive rounds of viral replication and passage.

One interesting finding of this study is the potential evolutionary relationship between PRRSV-2 nsp2 deletion variants and DVG generation. nsp2 contains a central hypervariable region that tolerates extensive deletions while maintaining viral viability, making it distinct from most regions of the PRRSV-2 genome. Here, we found that nsp2 represented a major hotspot for DVGs arising from within the same gene, and naturally occurring nsp2 deletion boundaries shared several features associated with DVG generation, including AU-rich junction regions, short homologous sequences, and overlapping break and rejoin sites with some DVGs. Overall, these results suggest that naturally occurring nsp2 deletions and DVGs may arise through similar template-switching mechanisms during viral RNA replication. Similar phenomena have been reported in other RNA viruses. For instance, in the fungal hypovirus VpHV1, internally deleted variants (VpHV1-β and VpHV1-γ) emerged during serial passage and retained independent replication competence without requiring a helper virus [64]. Although the biological background differs substantially from that of PRRSV-2, these observations demonstrate that deletion-containing viral genomes generated through RNA recombination can, under certain circumstances, persist and become evolutionarily stable forms. Based on these findings, we propose that DVG-associated template-switching events may occasionally generate deletion-containing PRRSV-2 genomes that remain replication competent. If such genomes retain sufficient fitness, they could subsequently be maintained through viral replication and evolutionary selection. This mechanism may partially explain the recurrent and independent emergence of nsp2 deletion variants in multiple PRRSV-2 lineages, including HP-PRRSV, NADC30-like, and NADC34-like strains. Given that PRRSV-2 nsp2 plays important roles in viral replication, immune modulation, and host adaptation [65–67], deletion events arising within this region may contribute to viral genome plasticity and potentially influence long-term viral evolution. Nevertheless, the present study provides only indirect evidence for such a mechanism. Future studies combining reverse genetics and long-read sequencing will be required to determine whether DVG-related recombination events directly contribute to the generation and maintenance of naturally occurring nsp2 deletion variants.

More importantly, we confirmed that naturally occurring PRRSV-2 DVGs possess potent antiviral activity. Using engineered DVG plasmids, we demonstrated that these DVGs effectively inhibit PRRSV-2 or PRRSV-1 replication. Notably, this interference was observed not only across multiple PRRSV-2 strains but also extended to PRRSV-1, suggesting a potential for broad-spectrum antiviral activity against PRRSVs. Consistent with our findings, previous studies have shown that DVGs can interfere with viral replication through diverse mechanisms in multiple RNA viruses and often exhibit broad-spectrum antiviral properties [34–36,57]. A key question for future research is whether PRRSV-2 DVGs can exert antiviral effects in mammalian *in vivo* models. Given the limited cross-protection of current attenuated PRRSV-2 vaccines and the lack of broad-spectrum antiviral drugs, DVG-based antiviral strategies hold promise as a complementary or potentially alternative approach to conventional vaccination.

Several key questions remain to be addressed in future studies. For example, it is still unclear whether the antiviral activity of PRRSV-2 DVGs primarily relies on competition with the full-length viral genome during replication and packaging, or relies on activation of innate immune responses. In addition, the molecular basis underlying the observed differences in DVG generation and accumulation across cell types and viral genotypes remains to be elucidated. Further investigation into how host background and viral genetic variation jointly shape DVG heterogeneity will provide deeper insights into their formation mechanisms. Finally, the dynamics of DVG production during serial viral passages remain poorly understood, and related studies are needed to clarify their formation, amplification, and selection during infection.

In summary, this study provides the first comprehensive characterization of PRRSV-2 DVGs and demonstrates that DVGs are commonly generated during PRRSV-2 infection, with their formation closely associated with microhomology sequences, RNA secondary structure, and AU-rich sequences. Notably, we identified a potential association between DVG generation and naturally occurring nsp2 deletion variants, suggesting that DVG-associated recombination may contribute to nsp2 genome evolution. In addition, PRRSV-2 DVGs exhibited broad-spectrum antiviral activity against multiple prevalent viral lineages. Together, these findings advance our understanding of DVG formation mechanisms, PRRSV-2 evolution, and the potential application of DVGs in antiviral strategies.

## Materials and methods

### Cells and viruses

Marc-145 cells (African green monkey kidney epithelial cells, ATCC CRL-12231) were obtained from the American Type Culture Collection (ATCC) and cultured in Dulbecco’s modified Eagle’s medium (DMEM) (Sigma-Aldrich, USA) supplemented with 10% fetal bovine serum, 100 μg/mL of streptomycin, and 100 IU/mL of penicillin at 37°C with 5% CO_2_. The HP-PRRSV HuN4 strain (GenBank accession no. EF635006.1), NADC30-like HeB108 strain (GenBank accession no. MN046224.1), NADC34-like HLJ29 strain, and PRRSV-1 DV strain were stored in our laboratory.

### Dataset preparation

To investigate DVG generation during PRRSV-2 infection, we searched for available RNA-seq datasets from the NCBI BioProject database until 1 March 2026. The search was restricted to studies that used *in vitro* cell samples and *in vivo* tissue samples infected with PRRSV-2. Finally, a total of 24 RNA-seq samples were selected for downstream analysis, with detailed information on the virus strain, MOI, infection time, DOI, and corresponding lineage provided in S2 Table.

### Sequence data processing and analysis

Raw reads were first trimmed for low-quality bases and adaptors using Trimmomatic (v0.39) with default parameters, and reads shorter than 36 bp were discarded. The high-quality reads were then aligned to the host reference genome using Bowtie2 (v2.2.9). Unmapped reads from the alignment were subsequently analyzed using the virus recombination mapper (ViReMa, v0.25) algorithm to report deletion events. ViReMa was run with parameters described previously [47], generating an output file “Virus_Recombination_Results.txt” that records the break and rejoin position coordinates of each junction read. ViReMa output files were further processed and filtered using criteria adapted from a previous study [52]. Specifically, (i) only junction reads with deletion sizes greater than 25 nt were retained to minimize false positives caused by short-range mismatches or sequencing errors; and (ii) both the break and rejoin positions were required to fall within coding regions downstream of the first 230 nt of the viral genome, to exclude interference from TRS-L-mediated events. Finally, DVG reads were classified as −sense DVG or +sense DVG according to the strand information reported in the ViReMa output. As PRRSV-2 is a positive-sense single-stranded RNA virus, all subsequent DVG analyses were performed using −sense DVGs, and only samples containing more than 50 unique DVG junction events were included.

### Characteristic analysis of DVGs

To investigate the potential role of repeated sequences in DVG formation, we utilized a custom Python script to analyze the homology at DVG break and rejoin positions across different PRRSV-2 strains. For each identified DVG, we quantified the number of identical nucleotides flanking the break and rejoin positions within defined upstream and downstream windows, allowing assessment of local microhomology associated with recombination events. Microhomology was defined as stretches of at least four identical nucleotides located near the break and rejoin sites, positioned at the same location or offset by a single nucleotide [30]. For regions longer than four nucleotides, a single internal mismatch was tolerated if at least two consecutive identical nucleotides were present on each side, in which case the entire region was still considered a microhomologous segment. Furthermore, adenosine plus uracil (AU) and guanine plus cytosine (GC) contents at each position were calculated using the R package *Biostrings* [68].

### RNA secondary structure and DVG junction distance analysis

RNA secondary structure analysis was performed to investigate the structural context of DVG break and rejoin sites. DVGs with deletion lengths ≤100 nt were selected for analysis because RNAfold (v2.4.17) has limitations in accurately predicting the secondary structures of very long RNA sequences [54]. For each DVG, genomic sequences spanning ±20 nt around both break and rejoin sites were extracted from the reference PRRSV-2 genome. RNA secondary structures were predicted using RNAfold (v2.4.17), and minimum free energy (MFE) structures were obtained. The resulting dot-bracket notation was used to construct RNA secondary structure graphs, in which nucleotides were represented as nodes, and both backbone connections and base-pairing interactions were represented as edges. Structural distance between break and rejoin sites was calculated as the shortest path length between corresponding nucleotides within the RNA structure graph using the *igraph* package in R. To assess whether observed structural distances deviated from random expectations, a permutation-based null model was generated by randomly sampling nucleotide positions within each RNA structure while preserving sequence length. Structural distances for random pairs were calculated using the same graph-based approach. The custom scripts used in this study are provided in S1 Text.

### Detection of DVG

To validate the presence of DVGs, a forward primer P1 (840F, 5′-TTGAAGTTGGTTGCGAACCG-3′) and two reverse primers, P2 (13855R, 5′-GTGCCATTCAGCTCACATAG-3′) and P3 (14541R, 5′-TACACTCCCCAAAGAAGTGC-3′), were designed to specifically amplify DVGs with deletion ranges of 1,065–13,652 and 1,065–14,341 nt, respectively. Total RNA was extracted from PRRSV-2-infected cell lysates using the RNA Easy Fast Tissue/Cell Kit (Tiangen, Beijing, China) according to the manufacturer’s instructions. RT-PCR products corresponding to both DVGs were approximately 400 bp in size and were separated on 1% agarose gels, stained with ethidium bromide, and visualized under UV illumination. The target bands were excised and purified using the Gel Extraction Kit (Omega, USA) and subsequently submitted to RuiBiotech Co., Ltd. (Harbin, China) for Sanger sequencing.

To assess DVG1 RNA replication in the presence or absence of WT PRRSV-2, RNA levels of DVG1 and the control plasmid were quantified by RT-qPCR. For DVG1 detection, primers and a TaqMan probe were designed based on the DVG1 sequence, with the probe targeting the deletion junction to specifically detect DVG1 RNA. The sequences of the primers and probe are listed in S3 Table. The control plasmid contains a PRRSV-1-derived M gene sequence, and its RNA level was detected using previously published primers and probe [69]. RT-qPCR was performed using the Premix Ex Taq (Probe qPCR) Kit (TaKaRa, Dalian, China) on a QuantStudio 5 Real-Time PCR System (Applied Biosystems), according to the manufacturer’s instructions. The copy numbers of DVG1 RNA were calculated based on standard curves generated using serially diluted plasmids containing the corresponding target sequence.

### Construction of PRRSV-2 DVG clones

DVG-encoding clones were derived from the PRRSV-2 HuN4 infectious clone using primers to excise the deleted region. The designed deletions corresponded to the following genomic regions (nt): 1,065–14,341 for DVG1; 1,065–13,652 for DVG2. The resulting DVG clones were verified by Sanger sequencing, and high-quality plasmid DNA was prepared using the PureLink HiPure Plasmid Midiprep Kit (Invitrogen, USA) for subsequent cell transfection and functional assays.

### *In vitro* DVG antiviral assay

To evaluate the antiviral activity of DVGs *in vitro*, Marc-145 cells were transfected with DVG plasmids or empty vector using Lipofectamine 3000 (Invitrogen, USA). At 24 hours post-transfection (hpt), the medium was replaced with fresh culture medium, and cells were subsequently infected with PRRSV-2 HuN4 strain at an MOI of 0.1. At 48 hpi, culture supernatants were collected for viral titer determination.

### Detection of antiviral gene mRNA by RT-qPCR

To determine whether DVGs induce antiviral gene expression, total RNA was extracted from infected cells using the Simply P Total RNA Extraction Kit (BioFlux, Hangzhou, China), and cDNA was synthesized using MLV reverse transcriptase with random primers (TaKaRa, Beijing, China). Quantitative PCR was then performed with gene-specific primers (S3 Table) using a ChamQ Universal SYBR qPCR Master Mix (Vazyme, Nanjing, China). Gene expression levels were normalized to *GAPDH* and relative fold changes were calculated using the ΔΔCt method, compared to mock-treated controls. All experiments were conducted in biological triplicates.

### Statistical analysis

All statistical analyses were performed using R version 4.2.1 or GraphPad Prism 10.0 (GraphPad Software, Inc.). For comparisons between two groups, a two-tailed Student’s t-test was used, with paired or unpaired tests selected according to the experimental design. The two-sided Wilcoxon rank-sum test was used for comparisons between two independent groups when the data did not meet the assumptions of parametric tests, whereas the two-sided Wilcoxon signed-rank test was used for paired comparisons. For comparisons involving more than two groups with a single experimental factor, one-way analysis of variance (ANOVA) followed by Dunnett’s multiple-comparisons test was used. For experiments involving two experimental factors and more than two groups, two-way ANOVA followed by Sidak’s multiple-comparisons test was used.

## Supporting information

S1 Fig (A–C) Kernel density analysis of DVG deletion lengths generated by different PRRSV-2 strains in PAMs (A), Marc-145 cells (B), and lung tissue (C). Each point represents a DVG supporting an individual deletion, and the y-axis represents the abundance of DVG. In addition, the extension of the x-axis in the KDE curve to negative length values is a smoothing artifact and has no biological significance.
(TIF)

S2 Fig (A) Kernel density estimation of DVG sizes derived from all strains, with the local minimum used as the threshold separating short DVGs from long DVGs. (B) Proportion of short DVGs and long DVGs produced by each strain in cells or tissue based on the DVG length classification. (C) Comparison of short and long DVG production between PAM cells, Marc-145 cells and tissue. Error bars represent the mean ± SD. Statistical significance was assessed using a two-tailed paired Student’s t-test. ns, not significant; *, *P* < 0.05.
(TIF)

S3 Fig (A–C) Distribution of DVG break and rejoin positions generated from PAMs (A), Marc-145 cells (B), and lung tissue (C). Each dot represents an individual DVG, plotted by its break position (y-axis) and rejoin position (x-axis), with color intensity indicating its relative abundance.
(TIF)

S4 Fig (A) The sequences of the amplification products generated by the P1/P2 and P1/P3 primer pairs were mapped to the HuN4 genome. Samples 1–3 were amplified with DVG 1065_13652 primers and originated from input virus, virus-infected cell culture supernatant, and virus-infected cell lysate, respectively. Samples 5–7 were amplified with DVG 1065_14341 primers in the same order. (B) Sequences from samples 1–3 and 5–7. The sequences at the break positions are shown in black, the sequences at the rejoin positions are in red, and the colons (:) in the middle indicate the presence of deletions. The superscript numbers above the nucleotides indicate the break and rejoin sites.
(TIF)

S1 Table Viral and DVG reads identified from PRRSV-2 RNA-seq data using ViReMa. (XLSX)

S2 Table Information on PRRSV-2 RNA-seq from public databases used in this study. (XLSX)

S3 Table Primers used in this study. (DOCX)

S4 Table DVG junction dataset used for analysis in this study. (XLSX)

S5 Table Source data for the experiments. (XLSX)

S1 Text Custom analysis and visualization scripts used in this study. (PDF)

S1 File Raw gel. Raw gel image from the study. (PDF)
