# Supplementary material for: Defective viral genomes of porcine reproductive and respiratory syndrome virus type 2: Generation, characterization and interference effects on viral replication
Source: PLoS Pathog. 2026 Oct 28;22(10):e1014755. doi: 10.1371/journal.ppat.1014755 (PMC13734580; doi:10.1371/journal.ppat.1014755)
Supplement: Supplementary file 1 [file ppat.1014755.s010.pdf]

1.Custom scripts

```
#!/usr/bin/env python3

"""
Microhomology analysis of DVG junction sequences.

This script identifies the number of matching nucleotides between the
5'-terminal sequences flanking the DVG break and rejoin sites. The sequence
to the left of the "|" delimiter is extracted from both reference sequences
and reversed before comparison.

The analysis allows a user-defined number of mismatches (default: 1).

Input:
    A tab-delimited DVG file containing at least 12 columns, including:
    Reference, Start, End, Type, Counts, Strand, RefStart, RefEnd,
    5'Ref, 3'Ref, Deletion_length, and Jfreq.

Output:
    A tab-delimited file containing the original columns plus MatchCount.
"""


def find_longest_homologous(seq1, seq2, max_mismatch=1):
    """
    Calculate the number of matching nucleotides between two sequences,
    allowing up to `max_mismatch` mismatches.

    Rules:
    1. If there are no mismatches, return the total number of matching
       nucleotides.
    2. If there is one mismatch:
       - Calculate the number of consecutive matching nucleotides
         immediately upstream and downstream of the mismatch.
       - If both sides contain at least 2 consecutive matching
         nucleotides, sum the matching nucleotides on both sides.
       - If both sides do not simultaneously contain at least 2
         consecutive matching nucleotides, return the larger of the
         two matching lengths.
    3. Stop the comparison when the second mismatch is encountered.

    Parameters
    ----------
    seq1 : str
        The first sequence.
    seq2 : str
        The second sequence.
    max_mismatch : int, default=1
        Maximum number of allowed mismatches.

    Returns
    -------
    int
        The number of matching nucleotides determined according to
```

```python
        the rules above.

    Raises
    ------
    ValueError
        If the two sequences have different lengths.
    """

    if len(seq1) != len(seq2):
        raise ValueError("The two sequences must have the same length.")

    n = len(seq1)
    mismatch_positions = []

    # Compare the two sequences nucleotide by nucleotide
    for i in range(n):
        if seq1[i] != seq2[i]:
            mismatch_positions.append(i)

            # Stop when the number of mismatches exceeds the allowed limit
            if len(mismatch_positions) > max_mismatch:
                break

    # ==========================================================
    # No mismatch
    # ==========================================================
    if len(mismatch_positions) == 0:
        return n

    # ==========================================================
    # One mismatch
    # ==========================================================
    mismatch_pos = mismatch_positions[0]

    # Calculate consecutive matching nucleotides upstream
    # of the mismatch
    left_match = 0
    j = mismatch_pos - 1

    while j >= 0 and seq1[j] == seq2[j]:
        left_match += 1
        j -= 1

    # Calculate consecutive matching nucleotides downstream
    # of the mismatch
    right_match = 0
    j = mismatch_pos + 1

    while j < n and seq1[j] == seq2[j]:
        right_match += 1
        j += 1

    # ==========================================================
    # Determine the matching length
    # ==========================================================
    if left_match >= 2 and right_match >= 2:
        # Both sides contain at least 2 consecutive matches
        match_count = left_match + right_match
```

```
    else:
        # Otherwise, use the longer matching segment
        match_count = max(left_match, right_match)

    return match_count


def extract_left_sequence(sequence):
    """
    Extract and reverse the sequence located to the left of "|".

    Parameters
    ----------
    sequence : str
        Sequence containing a "|" delimiter, for example:
        "GCCATGCTAACCAATTTGGC|TACCAAACCAAGCATGGTGT".

    Returns
    -------
    str
        Reversed sequence located to the left of the "|" delimiter.
    """

    print(f"\nExtracting the sequence to the left of '|': {sequence}")

    left_sequence = sequence.split("|")[0]

    print(f"Extracted sequence: {left_sequence}")

    reversed_sequence = left_sequence[::-1]

    print(f"Reversed sequence: {reversed_sequence}")

    return reversed_sequence


def process_file(input_file, output_file, max_mismatch=1):
    """
    Process a DVG file and calculate microhomology for each DVG junction.

    Parameters
    ----------
    input_file : str
        Path to the input tab-delimited DVG file.
    output_file : str
        Path to the output file.
    max_mismatch : int, optional
        Maximum number of mismatches allowed during sequence comparison.
        Default is 1.
    """

    with open(input_file, "r") as infile, open(output_file, "w") as outfile:

        # Write the output header.
        outfile.write(
            "Reference\t"
            "Start\t"
            "End\t"
```

```
            "Type\t"
            "Counts\t"
            "Strand\t"
            "RefStart\t"
            "RefEnd\t"
            "5'Ref\t"
            "3'Ref\t"
            "Deletion_length\t"
            "Jfreq\t"
            "MatchCount\n"
        )

        # Process each input line.
        for line_num, line in enumerate(infile, start=1):

            print(f"\nProcessing line {line_num}: {line.strip()}")

            # Remove the newline character and split the line by tabs.
            fields = line.strip().split("\t")

            print(f"Parsed fields: {fields}")

            # Check whether the input line contains enough columns.
            if len(fields) < 12:
                print(
                    f"Warning: Line {line_num} contains fewer than 12 columns "
                    f"and will be skipped."
                )
                continue

            # Extract the sequence fields.
            ref_sequence_field = fields[8]
            query_sequence_field = fields[9]

            print(f"Reference sequence field: {ref_sequence_field}")
            print(f"Query sequence field: {query_sequence_field}")

            # Extract the sequence to the left of "|" and reverse it.
            ref_sequence = extract_left_sequence(ref_sequence_field)
            query_sequence = extract_left_sequence(query_sequence_field)

            # Calculate the number of matching nucleotides.
            match_count = find_longest_homologous(
                ref_sequence,
                query_sequence,
                max_mismatch=max_mismatch
            )

            print(f"Match count: {match_count}")

            # Write the original fields and the calculated MatchCount.
            outfile.write(
                "\t".join(fields[:12])
                + f"\t{match_count}\n"
            )

            print(
                f"Result written for line {line_num}: "
```

```python
                f"{fields[:12]} + MatchCount = {match_count}"
            )

if __name__ == "__main__":

    # Specify the input and output files here.
    input_file = "input_DVG_data.txt"
    output_file = "DVG_microhomology_results.txt"

    # Set the maximum number of mismatches allowed.
    max_mismatch = 1

    print("Starting microhomology analysis...")

    process_file(
        input_file=input_file,
        output_file=output_file,
        max_mismatch=max_mismatch
    )

    print(
        f"\nAnalysis completed successfully. "
        f"Results were saved to: {output_file}"
    )
```

```r
# ============================================================
# RNA secondary structure and structural distance analysis
# of DVG break and rejoin sites
#
# This script:
# 1. Extracts local RNA sequences surrounding DVG break and
#    rejoin sites.
# 2. Predicts RNA secondary structures using RNAfold.
# 3. Identifies whether the break and rejoin sites are located
#    in stem or loop regions.
# 4. Calculates the shortest structural distance between the
#    break and rejoin sites based on the predicted secondary
#    structure.
#
# DVGs with deletion lengths <100 nt are analyzed.
# ============================================================


# ------------------------------------------------------------
# Load required packages
# ------------------------------------------------------------

library(Biostrings)
library(dplyr)
library(igraph)


# ------------------------------------------------------------
# Input DVG data
# ------------------------------------------------------------
```

```r
dvg_file <- "all.txt"

delVGs <- read.table(
  dvg_file,
  header = FALSE,
  sep = "\t",
  col.names = c(
    "name",
    "Break_Point",
    "Rejoin_Point",
    "Type",
    "counts",
    "strand",
    "reads_5",
    "reads_3",
    "seq_5",
    "seq_3",
    "Deletion_length",
    "Jfreq"
  )
)

head(delVGs)


# ------------------------------------------------------------
# Load the reference genome
# ------------------------------------------------------------

reference_file <- "PRRSV_Genome_SD16.fa"

ref <- readDNAStringSet(reference_file)

genome <- ref[[1]]

genome_length <- length(genome)


# ------------------------------------------------------------
# Extract local sequences surrounding DVG break and rejoin sites
#
# A 20-nt flanking region is extracted on both sides of the
# DVG junction:
#
#     Rejoin site
#          |
#     ----[20 nt]---|---[deletion]---|---[20 nt]----
#                                    Break site
#
# The extracted sequence is used for local RNA secondary
# structure prediction.
# ------------------------------------------------------------

delVGs$structure_seq <- sapply(
  seq_len(nrow(delVGs)),
  function(i) {
```

```
    rejoin <- delVGs$Rejoin_Point[i]
    break_point <- delVGs$Break_Point[i]

    start <- max(1, rejoin - 20)
    end <- min(genome_length, break_point + 20)

    as.character(
      subseq(
        genome,
        start = start,
        end = end
      )
    )
  }
)


# ------------------------------------------------------------
# Write sequences for RNAfold
# ------------------------------------------------------------

structure_fasta <- "DVG_structure.fa"

writeLines(
  paste0(
    ">DVG_",
    seq_len(nrow(delVGs)),
    "\n",
    delVGs$structure_seq
  ),
  structure_fasta
)


# ------------------------------------------------------------
# Predict RNA secondary structures using RNAfold
#
# RNAfold must be installed and available in the system PATH.
# ------------------------------------------------------------

structure_output <- "DVG_structure.out"

system(
  paste(
    "RNAfold <",
    structure_fasta,
    ">",
    structure_output
  )
)


# ------------------------------------------------------------
# Parse RNAfold output
# ------------------------------------------------------------

lines <- readLines(structure_output)
```

```r
# Identify lines containing dot-bracket structures.
structure_lines <- lines[grep("\\(", lines)]


# Extract dot-bracket structures.
dot_structure <- sub(
  " .*",
  "",
  structure_lines
)


# Extract minimum free energy (MFE).
mfe <- sub(
  ".*\\(([-0-9.]+)\\)",
  "\\1",
  structure_lines
)

mfe <- as.numeric(mfe)


# Add secondary structure and MFE to the DVG data frame.
delVGs$structure <- dot_structure
delVGs$MFE <- mfe


# -------------------------------------------------------------
# Define the positions of the rejoin and break sites within
# the extracted local sequences
#
# Because 20 nt upstream of the rejoin site are included,
# the rejoin site corresponds to position 21.
#
# The break site is located downstream by the deletion length.
# -------------------------------------------------------------

delVGs$rejoin_pos <- 21

delVGs$break_pos <- (
  21 +
    delVGs$Break_Point -
    delVGs$Rejoin_Point
)


# -------------------------------------------------------------
# Classify the secondary structure surrounding each junction
#
# "." represents an unpaired nucleotide (loop region),
# whereas "(" and ")" represent paired nucleotides
# (stem region).
# -------------------------------------------------------------

get_structure_type <- function(structure, position) {

  structure_vector <- strsplit(
```

```
    structure,
    ""
  )[[1]]

  if (position > length(structure_vector)) {
    return(NA_character_)
  }

  if (structure_vector[position] == ".") {
    return("loop")
  } else {
    return("stem")
  }
}


# Determine the structural context of the rejoin sites.
delVGs$rejoin_type <- mapply(
  get_structure_type,
  delVGs$structure,
  delVGs$rejoin_pos
)


# Determine the structural context of the break sites.
delVGs$break_type <- mapply(
  get_structure_type,
  delVGs$structure,
  delVGs$break_pos
)


# Summarize structural contexts.
table(delVGs$rejoin_type)

table(delVGs$break_type)


# ------------------------------------------------------------
# Calculate structural distance between DVG break and rejoin
# sites
#
# The RNA secondary structure is represented as an undirected
# graph containing:
#
# 1. Backbone connections between adjacent nucleotides.
# 2. Base-pair connections between paired nucleotides.
#
# The shortest path between the rejoin and break sites is then
# calculated using igraph.
# ------------------------------------------------------------

calc_structure_distance <- function(
  structure,
  position_1,
  position_2
) {
```

```r
structure_length <- nchar(structure)

# Initialize the edge list.
edges <- c()


# --------------------------------------------------------------
# Add backbone connections between adjacent nucleotides.
# --------------------------------------------------------------

if (structure_length > 1) {

  for (i in seq_len(structure_length - 1)) {

    edges <- c(
      edges,
      i,
      i + 1
    )
  }
}


# --------------------------------------------------------------
# Identify base-pairing relationships from dot-bracket
# notation.
# --------------------------------------------------------------

stack <- c()

paired_position <- rep(
  NA_integer_,
  structure_length
)


for (i in seq_len(structure_length)) {

  nucleotide_structure <- substr(
    structure,
    i,
    i
  )

  if (nucleotide_structure == "(") {

    stack <- c(
      stack,
      i
    )

  } else if (nucleotide_structure == ")") {

    paired_position_5prime <- tail(
      stack,
      1
    )
```

```r
    stack <- stack[
      -length(stack)
    ]

    paired_position[i] <-
      paired_position_5prime

    paired_position[paired_position_5prime] <-
      i
  }
}


# ----------------------------------------------------------
# Add base-pair connections.
# ----------------------------------------------------------

for (i in seq_len(structure_length)) {

  if (!is.na(paired_position[i])) {

    edges <- c(
      edges,
      i,
      paired_position[i]
    )
  }
}


# ----------------------------------------------------------
# Construct the RNA secondary structure graph.
# ----------------------------------------------------------

graph <- graph_from_edgelist(
  matrix(
    edges,
    ncol = 2,
    byrow = TRUE
  ),
  directed = FALSE
)


# ----------------------------------------------------------
# Calculate the shortest path between the rejoin and break
# sites.
# ----------------------------------------------------------

structural_distance <- distances(
  graph,
  v = position_1,
  to = position_2
)


return(
  as.numeric(structural_distance)
```

````
  )
}


# ------------------------------------------------------------
# Calculate the structural distance for each DVG.
# ------------------------------------------------------------

delVGs$structure_dist <- mapply(
  calc_structure_distance,
  delVGs$structure,
  delVGs$rejoin_pos,
  delVGs$break_pos
)


# ------------------------------------------------------------
# Save the results
# ------------------------------------------------------------

write.table(
  delVGs,
  file = "DVG_RNA_structure_analysis.txt",
  sep = "\t",
  row.names = FALSE,
  quote = FALSE
)


# ============================================================
# End of script
# ============================================================
```
````

```
# AU-content analysis of DVG break and rejoin junctions

library(Biostrings)

dat <- read.table(
  "input_DVG_data.txt",
  header = FALSE,
  sep = "\t"
)

dat$V9 <- gsub("\\|", "", dat$V9)
dat$V10 <- gsub("\\|", "", dat$V10)

rejoin_seq <- as.character(reverseComplement(DNAStringSet(dat$V10)))
break_seq  <- as.character(reverseComplement(DNAStringSet(dat$V9)))
reads <- as.numeric(dat$V5)

calculate_AU <- function(sequences, weights, n_pos = 40) {
  sapply(seq_len(n_pos), function(i) {
    bases <- substr(sequences, i, i)
    sum((bases == "A" | bases == "T") * weights) / sum(weights)
  })
```
```

```
}

rejoin_AU <- calculate_AU(rejoin_seq, reads)
break_AU  <- calculate_AU(break_seq, reads)

result <- data.frame(
  Position = seq_len(40),
  Rejoin_AU = rejoin_AU,
  Break_AU = break_AU
)

write.table(
  result,
  "NSP2_AU_content.txt",
  sep = "\t",
  col.names = TRUE,
  row.names = FALSE,
  quote = FALSE
)
```

2.Visualization code

```
#Fig 2A-D
library(ggplot2)
library(dplyr)
library(scales)
library(tidyr)

df <- read.table("DVG.txt", header=FALSE, sep="\t")
colnames(df) <- c("name","Rejoin_Point","Break_Point","Type","counts","strand",
                  "5'seq","3'seq","deletion_size","Jfreq")

df$rpm <- log10(df$Jfreq * 100 + 1)

dens <- density(df$deletion_size, na.rm=TRUE)
dens_df <- data.frame(deletion_size=dens$x, density=dens$y)

scale_factor <- max(df$rpm, na.rm=TRUE) / max(dens_df$density, na.rm=TRUE)

p <- ggplot() +
  geom_point(
    data=df,
    aes(x=deletion_size, y=rpm),
    position=position_jitter(width=5, height=0.05),
    shape=21, fill=NA, color="black", stroke=0.7, size=2, alpha=0.8
  ) +
  geom_area(
    data=dens_df,
    aes(x=deletion_size, y=density*scale_factor),
    fill="steelblue", alpha=0.3, colour=NA
  ) +
  scale_y_continuous(
    name=expression(Log[10] ~ "Reads per million"),
    sec.axis=sec_axis(
      ~ . / scale_factor,
      name="Density",
      labels=label_scientific(digits=2)
    )
  ) +
  scale_x_continuous(expand=c(0,0)) +
  theme_bw() +
  labs(title="Lung_SD53-1603", x="Deletion length (nt)") +
  theme(
    axis.text=element_text(size=22),
    axis.title=element_text(size=22),
    plot.title=element_text(hjust=0.5, size=30),
    text=element_text(family="sans")
  )

p

#Fig 2E-F
delVGs <- read.table("DVG.txt", header=FALSE, sep="\t")
colnames(delVGs) <-
c("name","Rejoin_Point","Break_Point","Type","counts","strand",
                  "5'seq","3'seq","deletion_size","Jfreq")
delVGs <- delVGs %>%
```

```
  mutate(Deletion_group = case_when(
    Deletion_length <= 4000 ~ "S-Del",
    Deletion_length > 4000 ~ "L-Del"
  ))
delVGs_prop <- delVGs %>%
  group_by(name, Deletion_group) %>%
  summarise(weighted_count=sum(counts,na.rm=TRUE),.groups="drop") %>%
  group_by(name) %>%
  mutate(prop=weighted_count/sum(weighted_count)) %>%
  ungroup()

ggplot(delVGs_prop,aes(x=name,y=prop,fill=Deletion_group)) +
  geom_col() +
  scale_y_continuous(labels=scales::label_percent()) +
  labs(x="Strain",y="Relative frequency (%)",fill="Deletion group") +
  theme_bw() +
  theme(axis.text.x=element_text(angle=45,hjust=1))

#Fig 3A
df <- read.table("DVG.txt",header=FALSE,sep="\t")
colnames(df) <- c("name","start","end","Type","counts","strand",
                  "reads_5","reads_3","seq_5","seq_3","deletion_size","Jfreq")

ggplot(df,aes(x=end,y=start)) +
  stat_summary_2d(aes(z=counts),bins=80,fun=sum) +
  geom_abline(slope=1,intercept=0,linetype="dashed",color="black") +
  scale_fill_gradientn(
    name="Counts",
    colors=c("#3B0F70","#F1605D","#FCFDBF"),
    values=scales::rescale(c(0,max(df$counts)/5,max(df$counts)))) +
  labs(title="PAM_YC2020",
       x="Stop (nucleotide position)",
       y="Start (nucleotide position)") +
  coord_cartesian(xlim=c(0,15000),ylim=c(0,15000)) +
  theme_bw() +
  theme(text=element_text(family="sans"),
        plot.title=element_text(hjust=0.5,size=30),
        legend.title=element_text(size=22),
        legend.text=element_text(size=22),
        axis.text=element_text(size=22),
        axis.title=element_text(size=22))

#Fig 3B
delVGs <- read.table("all_DVG.txt",header=FALSE,sep="\t",
  col.names=c("name","Break_Point","Rejoin_Point","Type","counts","strand",
              "reads_5","reads_3","seq_5","seq_3","Deletion_length","Jfreq"))
# Weighted KDE
dens_break <- density(delVGs$Break_Point,
  weights=delVGs$Jfreq/sum(delVGs$Jfreq),n=2000)
dens_rejoin <- density(delVGs$Rejoin_Point,
  weights=delVGs$Jfreq/sum(delVGs$Jfreq),n=2000)

df_combined <- rbind(
  data.frame(Position=dens_break$x,Density=dens_break$y,Type="Break"),
  data.frame(Position=dens_rejoin$x,Density=dens_rejoin$y,Type="Rejoin"))

# Genome structure
genome_structure <- data.frame(
```

```
  gene=c("5'UTR","ORF1a","ORF1b","GP2","GP3","GP4","GP5","M","N","3'UTR"),
  start=c(1,190,7612,11983,12606,13151,13698,14285,14799,15171),
  end=c(189,7611,11981,12753,13370,13687,14300,14809,15170,15358))

x_min <- min(genome_structure$start)
x_max <- max(genome_structure$end)

# Plot
p_density <- ggplot(df_combined,aes(x=Position,y=Density,color=Type)) +
  geom_line(linewidth=1) +
  scale_color_manual(values=c(Break="steelblue",Rejoin="firebrick")) +
  scale_x_continuous(limits=c(x_min,x_max)) +
  labs(x=NULL,y="Jfreq-weighted density",color="Point type") +
  theme_bw() +
  theme(axis.title=element_text(size=14),
        axis.text=element_text(size=12),
        legend.position="top",
        plot.margin=margin(t=5,r=5,b=0,l=5))

p_genome <- ggplot(genome_structure) +
  geom_rect(aes(xmin=start,xmax=end,ymin=0,ymax=0.5,fill=gene),color="black") +
  scale_fill_brewer(palette="Paired") +
  scale_x_continuous(limits=c(x_min,x_max)) +
  labs(x="Genome position",y=NULL) +
  theme_bw() +
  theme(axis.text.y=element_blank(),
        axis.ticks.y=element_blank(),
        axis.title.y=element_blank(),
        legend.position="bottom",
        plot.margin=margin(t=0,r=5,b=5,l=5))

p_combined <- p_density / p_genome + plot_layout(heights=c(3,0.25))
p_combined

#Fig 3C-D
delVGs <- read.table("all_DVG.txt",header=FALSE,sep="\t",
  col.names=c("name","Break_Point","Rejoin_Point","Type","counts","strand",
           "reads_5","reads_3","seq_5","seq_3","Deletion_length","Jfreq"))

group_map <- data.frame(
  name = c(
    # L8
    "PAM_SD16", "Marc_TA-12", "Marc_JXwn06", "PAM_WUH3",
    "PAM_GSWW15", "PAM_JXwn06", "PAM_JXA1", "Marc_HuN4",
    "Marc_HN07-1", "BALF_Li11", "Lung_S75", "Marc_SD16", "Marc_GD-HD", "PAM_GD-
HD",
    # L1
    "PAM_YC2020", "PAM_NC174", "Lung_HB94", "Lung_SD53-1603",
    # L5
    "Marc_R98","Marc_VR2332","PAM_FL12", "Lung_G101"
  ),

  lineage = c(
    rep("L8", 14),
    rep("L1", 4),
    rep("L5", 4)
  )
)
```

```
delVGs <- delVGs %>% left_join(group_map,by="name")

gene_info_L1 <- data.frame(
  Gene=c("5'UTR","NSP1","NSP2","NSP3","NSP4","NSP5","NSP6","NSP7",
         "NSP8","NSP9","NSP10","NSP11","NSP12","GP2","GP2/3","GP3",
         "GP3/4","GP4","GP5","M","N","3'UTR"),
  Start=c(1,191,1337,4526,5219,5831,6341,6389,7166,7291,9220,10543,
          11212,11675,12298,12446,12843,13063,13390,13977,14491,14863),
  End=c(190,1336,4525,5218,5830,6340,6388,7165,7300,9219,10542,11211,
        11670,12297,12445,12842,13062,13379,13992,14501,14862,15039))

gene_info_L8 <- data.frame(
  Gene=gene_info_L1$Gene,
  Start=c(1,190,1339,4834,5227,6139,6649,6697,7474,7599,9528,10851,
          11520,11983,12606,12754,13151,13371,13698,14285,14799,15171),
  End=c(189,1338,4833,5226,6138,6648,6696,7473,7611,9527,10850,11519,
        11981,12605,12753,13150,13370,13687,14300,14809,15170,15340))

gene_info_L5 <- data.frame(
  Gene=gene_info_L1$Gene,
  Start=c(1,192,1338,4926,5619,6231,6741,6789,7566,7691,9620,10943,
          11612,12075,12698,12846,13243,13463,13790,14377,14891,15263),
  End=c(191,1337,4925,5618,6230,6740,6788,7565,7700,9619,10942,11611,
        12073,12697,12845,13242,13462,13779,14392,14901,15262,15436))

annotate_breakpoint <- function(data,gene_info){
  data %>%
    rowwise() %>%
    mutate(Break_group=ifelse(
      any(Break_Point>=gene_info$Start & Break_Point<=gene_info$End),
      gene_info$Gene[which(Break_Point>=gene_info$Start &
                           Break_Point<=gene_info$End)[1]],
      "Intergenic")) %>%
    ungroup()
}

data_L1 <- delVGs %>% filter(lineage=="L1") %>%
annotate_breakpoint(gene_info_L1)
data_L8 <- delVGs %>% filter(lineage=="L8") %>%
annotate_breakpoint(gene_info_L8)
data_L5 <- delVGs %>% filter(lineage=="L5") %>%
annotate_breakpoint(gene_info_L5)

data_combined <- bind_rows(data_L1,data_L8,data_L5) %>%
  group_by(name,Break_group) %>%
  summarise(Jfreq_sum=sum(Jfreq,na.rm=TRUE),.groups="drop") %>%
  tidyr::pivot_wider(names_from=Break_group,values_from=Jfreq_sum,values_fill=0)
%>%
  rowwise() %>%
  mutate(Total=sum(c_across(-name),na.rm=TRUE)) %>%
  ungroup() %>%
  mutate(across(-c(name,Total),~.x/Total))

heatmap_matrix <- data_combined %>%
  tibble::column_to_rownames("name") %>%
  as.matrix() %>%
  t()
```

```r
heatmap_matrix <- heatmap_matrix[
  !rownames(heatmap_matrix)%in%c("Total","Intergenic"),]

custom_col_order <- c(
  "PAM_FL12","PAM_GSWW15","PAM_JXwn06","PAM_NC174","PAM_GD-HD",
  "PAM_SD16","PAM_WUH3","PAM_YC2020","PAM_JXA1","Marc_HuN4",
  "Marc_JXwn06","Marc_R98","Marc_VR2332","Marc_GD-HD","Marc_SD16",
  "Marc_TA-12","Marc_HN07-1","BALF_Li11","Lung_G101","Lung_HB94",
  "Lung_S75","Lung_SD53-1603")
heatmap_matrix <- heatmap_matrix[, custom_col_order]
mat_log <- log10(heatmap_matrix + 1)

p <- pheatmap(
  mat_log,
  cellwidth = 20,
  cellheight = 15,
  cluster_rows = TRUE,
  cluster_cols = FALSE,
  color = colorRampPalette(
    c("steelblue", "white", "#F6DB91", "#CF332A")
  )(100),
  display_numbers = FALSE,
  main = "Rejoin point"
)
p

#Fig 4B-C
library(tidyverse)
library(pheatmap)
delVGs <- read.table("homology.txt",
                     header = FALSE,
                     sep = "\t",
                     col.names = c("Reference", "Break_Point", "Rejoin_Point",
"Type", "counts", "strand","5'_read", "3'_read", "seq_5",
"seq_3","Deletion_length", "Jfreq", "MatchCount")
)

delVGs <- delVGs %>%
  mutate(Deletion_group = case_when(
    Deletion_length <= 4000 ~ "S-Del",
    Deletion_length > 4000 ~ "L-Del"
  ))

df_mc <- delVGs %>%
  group_by(Reference, Deletion_group, MatchCount) %>%
  summarise(TotalCounts = sum(Jfreq), .groups = "drop") %>%
  group_by(Reference, Deletion_group) %>%
  mutate(Percent = TotalCounts / sum(TotalCounts)) %>%
  ungroup()

df_L <- df_mc %>%
  filter(Deletion_group == "L-Del") %>%
  select(Reference, MatchCount, Percent) %>%
  pivot_wider(names_from = Reference, values_from = Percent, values_fill = 0)
df_L <- df_L[order(df_L$MatchCount), ]
mat_L <- as.matrix(df_L[,-1])
rownames(mat_L) <- df_L$MatchCount
```

```r
df_S <- df_mc %>%
  filter(Deletion_group == "S-Del") %>%
  select(Reference, MatchCount, Percent) %>%
  pivot_wider(names_from = Reference, values_from = Percent, values_fill = 0)
df_S <- df_S[order(df_S$MatchCount), ]
mat_S <- as.matrix(df_S[,-1])
rownames(mat_S) <- df_S$MatchCount

custom_col_order <- c("PAM_FL12", "PAM_GSWW15", "PAM_JXwn06", "PAM_NC174",
"PAM_GD-HD", "PAM_SD16", "PAM_WUH3",  "PAM_YC2020", "PAM_JXA1","Marc_HuN4",
"Marc_JXwn06", "Marc_R98", "Marc_GD-HD", "Marc_SD16", "Marc_TA-12",
"Marc_VR2332", "Marc_HN07-1",
"BALF_Li11", "Lung_G101", "Lung_HB94", "Lung_S75", "Lung_SD53-1603")

mat_L <- mat_L[, custom_col_order]
p1 <- pheatmap(
  mat_L,
  cluster_cols = FALSE,
  cluster_rows = FALSE,
  cellwidth = 20,
  cellheight = 20,
  color = colorRampPalette(
    c("steelblue", "white", "#F6DB91", "#CF332A")
  )(100),
  breaks = seq(0, 0.3, length.out = 101),
  legend_breaks = c(0, 0.10, 0.20, 0.30),
  legend_labels = c("0", "0.10", "0.20", "≥0.30"),
  display_numbers = FALSE,
  main = "L-Del"
)
p1

#Fig 4D
mh_cdf_sample <- delVGs %>%
  filter(Deletion_group %in%c("L-Del","S-Del"),
         MatchCount>=0,MatchCount<=20) %>%
  group_by(Reference,Deletion_group,MatchCount) %>%
  summarise(Jfreq=sum(Jfreq,na.rm=TRUE),.groups="drop") %>%
  group_by(Reference,Deletion_group) %>%
  complete(MatchCount=0:20,fill=list(Jfreq=0)) %>%
  arrange(MatchCount,.by_group=TRUE) %>%
  mutate(Cumulative=cumsum(Jfreq)/sum(Jfreq)) %>%
  ungroup()

sample_check <- mh_cdf_sample %>%
  distinct(Reference,Deletion_group) %>%
  count(Reference) %>%
  filter(n==2)

cdf_summary <- mh_cdf_sample %>%
  group_by(Deletion_group,MatchCount) %>%
  summarise(
    Mean=mean(Cumulative,na.rm=TRUE),
    SD=sd(Cumulative,na.rm=TRUE),
    N=sum(!is.na(Cumulative)),
    SE=SD/sqrt(N),
    Lower=Mean-1.96*SE,
```

```
    Upper=Mean+1.96*SE,
    .groups="drop"
  )

ggplot(cdf_summary,
       aes(x=MatchCount,y=Mean,color=Deletion_group,fill=Deletion_group)) +
  geom_ribbon(aes(ymin=Lower,ymax=Upper),alpha=0.15,color=NA) +
  geom_step(linewidth=1.3) +
  scale_x_continuous(breaks=0:19,limits=c(0,19),
                     expand=expansion(mult=c(0.01,0.02))) +
  scale_y_continuous(breaks=seq(0,1,0.2),
                     labels=scales::percent_format(accuracy=1),
                     limits=c(0,1),expand=c(0,0)) +
  labs(x="Microhomology length (nt)",
       y="Cumulative relative Jfreq (%)",
       color=NULL,fill=NULL) +
  theme_classic(base_size=14) +
  theme(axis.text=element_text(size=12,color="black"),
        axis.title=element_text(size=16,color="black"),
        legend.position="top",
        legend.text=element_text(size=13),
        legend.key.width=unit(1.5,"cm"),
        axis.ticks=element_line(color="black",linewidth=0.5),
        axis.ticks.length=unit(0.15,"cm"),
        plot.margin=margin(10,15,10,10),
        aspect.ratio=0.8)

# Fig 4E
mh_by_sample <- delVGs %>%
  filter(Deletion_group %in%c("L-Del","S-Del"),
         MatchCount>=0,MatchCount<=20) %>%
  group_by(Reference,Deletion_group,MatchCount) %>%
  summarise(Abundance=sum(Jfreq,na.rm=TRUE),.groups="drop") %>%
  complete(Reference,Deletion_group,MatchCount=0:20,
           fill=list(Abundance=0)) %>%
  group_by(Reference,Deletion_group) %>%
  mutate(Percent=Abundance/sum(Abundance)*100) %>%
  ungroup()

mh_distribution_summary <- mh_by_sample %>%
  group_by(Deletion_group,MatchCount) %>%
  summarise(
    N=sum(!is.na(Percent)),
    Mean=mean(Percent,na.rm=TRUE),
    SD=sd(Percent,na.rm=TRUE),
    SE=SD/sqrt(N),
    Lower=Mean-qt(0.975,df=N-1)*SE,
    Upper=Mean+qt(0.975,df=N-1)*SE,
    .groups="drop"
  )

ggplot(mh_distribution_summary,
       aes(x=MatchCount,y=Mean,color=Deletion_group,
           fill=Deletion_group,group=Deletion_group)) +
  geom_ribbon(aes(ymin=Lower,ymax=Upper),alpha=0.15,color=NA) +
  geom_line(linewidth=1.2) +
  geom_point(size=3) +
  scale_x_continuous(breaks=0:19,limits=c(0,19)) +
```

```r
  scale_y_continuous(limits=c(0,30),breaks=seq(0,30,5)) +
  labs(x="Microhomology length (nt)",
       y="Relative Jfreq (%)",
       color=NULL,fill=NULL) +
  theme_classic() +
  theme(axis.text=element_text(size=14),
        axis.title=element_text(size=16),
        legend.text=element_text(size=13),
        legend.position="top",
        aspect.ratio=0.8)

#Fig 5A
delVGs <- read.table("all_DVG.txt",header=FALSE,sep="\t",
  col.names=c("name","Break_Point","Rejoin_Point","Type","counts","strand",
              "reads_5","reads_3","seq_5","seq_3","Deletion_length","Jfreq"))

jfreq_sum <- delVGs %>%
  filter(Deletion_length<100) %>%
  group_by(name) %>%
  summarise(Jfreq=sum(Jfreq,na.rm=TRUE),.groups="drop") %>%
  arrange(desc(Jfreq))

ggplot(jfreq_sum,aes(x=reorder(name,Jfreq),y=Jfreq,fill=name)) +
  geom_col() +
  coord_flip() +
  labs(x="Sample name",y="Jfreq") +
  theme_bw() +
  theme(legend.position="none",
        axis.title=element_text(size=10),
        axis.text.x=element_text(size=14),
        axis.text.y=element_text(size=12),
        aspect.ratio=1.5)

#Fig 5D-E
set.seed(123)
random_structure_dist <- mapply(function(structure, L){
  n <- nchar(structure)
  pos1 <- sample(1:(n - L), 1)
  pos2 <- pos1 + L

  calc_structure_distance(structure, pos1, pos2)

}, delVGs$structure, delVGs$Deletion_length)

wilcox.test(delVGs$structure_dist, random_structure_dist)

df_plot <- data.frame(
  dist = c(delVGs$structure_dist, random_structure_dist),
  group = rep(c("Observed", "Random"), each = nrow(delVGs))
)
p <- wilcox.test(delVGs$structure_dist, random_structure_dist)$p.value
ggplot(df_plot, aes(x = group, y = dist, fill = group)) +

  geom_boxplot(width = 0.6, outlier.shape = NA, alpha = 0.8) +
  geom_jitter(width = 0.15, alpha = 0.3, size = 1) +

  scale_fill_manual(values = c("#D73027", "#4575B4")) +
```

```
  annotate("text", x = 1.5, y = max(df_plot$dist, na.rm=TRUE),
           label = paste0("p = ", signif(p,3)), size = 5) +

  theme_bw() +
  theme(
    legend.position = "none",
    axis.text = element_text(size = 16),
    axis.title = element_text(size = 18),
    aspect.ratio = 1
  ) +

  labs(
    x = "",
    y = "Structure distance"
  )

cor.test(delVGs$Deletion_length, delVGs$structure_dist, method = "pearson")
ggplot(delVGs, aes(x = Deletion_length, y = structure_dist)) +
  geom_point(aes(size = Jfreq), alpha = 0.5) +
  geom_smooth(method = "lm", color = "red") +
  theme_bw() +
  theme(axis.text = element_text(size = 16),
        axis.title = element_text(size = 17),
        legend.title = element_text(size = 16),
        legend.text = element_text(size = 14)
  )
summary(lm(structure_dist ~ Deletion_length, data = delVGs))

# Fig 5F
library(dplyr)
library(dbscan)
library(ggplot2)

df <- read.table("HP-PRRSV.txt",header=FALSE,sep="\t",skip=1,
  col.names=c("name","Break_Point","Rejoin_Point","Type","counts","strand",
              "reads_5","reads_3","seq_5","seq_3","Deletion_length","Jfreq"))
%>%
  filter(Deletion_length<100) %>%
  mutate(Break_Rejoin=paste(Rejoin_Point,Break_Point,sep="_"))

coords <- df[,c("Break_Point","Rejoin_Point")]
db <- dbscan(coords,eps=3,minPts=1)
df$cluster <- db$cluster

hotspot <- df %>%
  group_by(cluster) %>%
  summarise(
    Break=round(mean(Break_Point)),
    Rejoin=round(mean(Rejoin_Point)),
    Counts=sum(counts,na.rm=TRUE),
    Jfreq=sum(Jfreq,na.rm=TRUE),
    n_DVG=n_distinct(Break_Rejoin),
    n_sample=n_distinct(name),
    DVGs=paste(unique(Break_Rejoin),collapse="; "),
    Samples=paste(unique(name),collapse="; "),
    .groups="drop"
  ) %>%
  mutate(Hotspot=paste(Rejoin,Break,sep="_")) %>%
```

```r
  arrange(desc(n_DVG),desc(Counts))

top10_plot <- hotspot %>%
  slice_head(n=10) %>%
  filter(!Hotspot %in%c("15111_15142","14349_14377",
                        "14839_14875","14029_14059"))

plot_df <- df %>%
  filter(cluster %in% top10_plot$cluster) %>%
  mutate(
    DVG=paste(Rejoin_Point,Break_Point,sep="_"),
    Sample=name
  ) %>%
  left_join(top10_plot %>% select(cluster,Hotspot),by="cluster") %>%
  group_by(Hotspot,DVG,Sample) %>%
  summarise(Jfreq=sum(Jfreq,na.rm=TRUE),.groups="drop")

ggplot(plot_df,aes(x=Sample,y=DVG,fill=Jfreq)) +
  geom_tile(colour="white") +
  facet_wrap(~Hotspot,scales="free_y",ncol=2) +
  scale_fill_viridis_c(name="Jfreq",trans="sqrt") +
  theme_bw() +
  theme(axis.text.x=element_text(angle=45,hjust=1),
        axis.title=element_blank(),
        legend.text=element_text(size=6),
        legend.title=element_text(size=8),
        aspect.ratio=0.4)

# Fig 7A
library(dplyr)
library(ggplot2)

delVGs <- read.table("all.txt",header=FALSE,sep="\t",
  col.names=c("name","Break_Point","Rejoin_Point","Type","counts","strand",
              "reads_5","reads_3","seq_5","seq_3","Deletion_length","Jfreq"))

group_map <- data.frame(
  name=c("PAM_SD16","Marc_TA-12","Marc_JXwn06","PAM_WUH3","PAM_GSWW15",
         "PAM_JXwn06","PAM_JXA1","Marc_HuN4","Marc_HN07-1","BALF_Li11",
         "Lung_S75","Marc_SD16","PAM_GD-HD","Marc_GD-HD",
         "PAM_YC2020","PAM_NC174","Lung_HB94","Lung_SD53-1603",
         "Marc_R98","Marc_VR2332","PAM_FL12","Lung_G101"),
  lineage=c(rep("L8",14),rep("L1",4),rep("L5",4))
)

gene_L8 <- data.frame(
  Gene=c("5'UTR","NSP1","NSP2","NSP3","NSP4","NSP5","NSP6","NSP7",
         "NSP8","NSP9","NSP10","NSP11","NSP12","GP2","GP2/3","GP3",
         "GP3/4","GP4","GP5","M","N","3'UTR"),
  Start=c(1,190,1339,4834,5227,6139,6649,6697,7474,7599,9528,10851,
          11520,11983,12606,12754,13151,13371,13698,14285,14799,15171),
  End=c(189,1338,4833,5226,6138,6648,6696,7473,7611,9527,10850,11519,
        11981,12605,12753,13150,13370,13687,14300,14809,15170,15340)
)

gene_L1 <- data.frame(
  Gene=c("5'UTR","NSP1","NSP2","NSP3","NSP4","NSP5","NSP6","NSP7",
         "NSP8","NSP9","NSP10","NSP11","NSP12","GP2","GP2/3","GP3",
```

```
        "GP3/4","GP4","GP5","M","N","3'UTR"),
  Start=c(1,191,1337,4526,5219,5831,6341,6389,7166,7291,9220,10543,
          11212,11675,12298,12446,12843,13063,13390,13977,14491,14863),
  End=c(190,1336,4525,5218,5830,6340,6388,7165,7300,9219,10542,11211,
        11670,12297,12445,12842,13062,13379,13992,14501,14862,15039)
)

gene_L5 <- data.frame(
  Gene=c("5'UTR","NSP1","NSP2","NSP3","NSP4","NSP5","NSP6","NSP7",
         "NSP8","NSP9","NSP10","NSP11","NSP12","GP2","GP2/3","GP3",
         "GP3/4","GP4","GP5","M","N","3'UTR"),
  Start=c(1,192,1338,4926,5619,6231,6741,6789,7566,7691,9620,10943,
          11612,12075,12698,12846,13243,13463,13790,14377,14891,15263),
  End=c(191,1337,4925,5618,6230,6740,6788,7565,7700,9619,10942,11611,
        12073,12697,12845,13242,13462,13779,14392,14901,15262,15436)
)

assign_gene <- function(pos,gene_df) {
  gene_df$Gene[match(TRUE,pos>=gene_df$Start & pos<=gene_df$End)]
}

process_lineage <- function(data,gene_df) {
  data %>%
    mutate(
      Break_gene=sapply(Break_Point,assign_gene,gene_df=gene_df),
      Rejoin_gene=sapply(Rejoin_Point,assign_gene,gene_df=gene_df)
    ) %>%
    filter(!is.na(Break_gene),
           !is.na(Rejoin_gene),
           Break_gene==Rejoin_gene) %>%
    group_by(name,Break_gene) %>%
    summarise(Jfreq=sum(Jfreq,na.rm=TRUE),.groups="drop") %>%
    group_by(name) %>%
    mutate(freq=Jfreq/sum(Jfreq)) %>%
    ungroup()
}

delVGs <- delVGs %>%
  left_join(group_map,by="name")

data_L1 <- process_lineage(filter(delVGs,lineage=="L1"),gene_L1)
data_L5 <- process_lineage(filter(delVGs,lineage=="L5"),gene_L5)
data_L8 <- process_lineage(filter(delVGs,lineage=="L8"),gene_L8)

data_combined <- bind_rows(data_L1,data_L5,data_L8)

nsp_order <- c("NSP1","NSP2","NSP3","NSP4","NSP5","NSP6",
               "NSP7","NSP8","NSP9","NSP10","NSP11","NSP12")

data_plot <- data_combined %>%
  filter(Break_gene %in%nsp_order) %>%
  mutate(Break_gene=factor(Break_gene,levels=nsp_order))

ggplot(data_plot,aes(x=name,y=Break_gene,fill=freq)) +
  geom_tile(color="white",linewidth=0.5) +
  scale_fill_gradient2(
    low="blue",mid="white",high="red",
    midpoint=median(data_plot$freq,na.rm=TRUE),
```

```r
    name="Relative Jfreq"
  ) +
  labs(x=NULL,y=NULL) +
  theme_bw() +
  theme(
    axis.text.x=element_text(angle=45,hjust=1,size=12),
    axis.text.y=element_text(size=12),
    axis.title=element_blank(),
    legend.position="right"
  )

#Fig 7B
delVGs_filtered <- delVGs %>%
  filter(
    (lineage == "L5" & Break_Point >= 1337 & Break_Point <= 4924 &
     Rejoin_Point >= 1337 & Rejoin_Point <= 4924) |
    (lineage == "L8" & Break_Point >= 1336 & Break_Point <= 4833 &
     Rejoin_Point >= 1336 & Rejoin_Point <= 4833) |
    (lineage == "L1" & Break_Point >= 1338 & Break_Point <= 4622 &
     Rejoin_Point >= 1338 & Rejoin_Point <= 4622)
  )

ggplot(delVGs_filtered,aes(x=Break_Point,y=Rejoin_Point)) +
  geom_bin2d(bins=60) +
  facet_wrap(~lineage,scales="free") +
  scale_fill_gradientn(
    name="Counts",
    colors=c("#3B0F70","#F1605D","#FCFDBF"),
    values=scales::rescale(c(
      0,
      max(delVGs_filtered$counts,na.rm=TRUE)/5,
      max(delVGs_filtered$counts,na.rm=TRUE)
    ))
  ) +
  labs(
    x="Break point (nucleotide position)",
    y="Rejoin point (nucleotide position)"
  ) +
  theme_bw() +
  theme(
    text=element_text(family="sans"),
    legend.title=element_text(size=18),
    legend.text=element_text(size=15),
    axis.text=element_text(size=18),
    axis.title=element_text(size=18),
    strip.text=element_text(size=18)
  )

#Fig 7C
delVGs_filtered_L5 <- delVGs_filtered %>%
  dplyr::filter(lineage == "L5")
nsp2_regions <- data.frame(
  region = c("Region1", "Region2", "Region3", "Region4"),
  start = c(1337, 2337, 3337, 4337),
  end = c(2336, 3336, 4336, 4924),
  description = c("N-terminal region", "Hypervariable region 1",
                  "Central region", "C-terminal region")
)
```

```r
dvg_region1 <- delVGs_filtered_L5 %>%
  filter(Break_Point >= 1337 & Break_Point <= 2336 &
           Rejoin_Point >= 1337 & Rejoin_Point <= 2336)

dvg_region2 <- delVGs_filtered_L5 %>%
  filter(Break_Point >= 2337 & Break_Point <= 3336 &
           Rejoin_Point >= 2337 & Rejoin_Point <= 3336)

dvg_region3 <- delVGs_filtered_L5 %>%
  filter(Break_Point >= 3337 & Break_Point <= 4336 &
           Rejoin_Point >= 3337 & Rejoin_Point <= 4336)

dvg_region4 <- delVGs_filtered_L5 %>%
  filter(Break_Point >= 4337 & Break_Point <= 4924 &
           Rejoin_Point >= 4337 & Rejoin_Point <= 4924)


dvg_by_region <- bind_rows(
  dvg_region1 %>% mutate(region = "Region1"),
  dvg_region2 %>% mutate(region = "Region2"),
  dvg_region3 %>% mutate(region = "Region3"),
  dvg_region4 %>% mutate(region = "Region4")
)

region_counts_weighted <- dvg_by_region %>%
  group_by(region, lineage) %>%
  summarise(
    total_weight = sum(counts, na.rm = TRUE),
    count = n(),
    .groups = "drop"
  )
```
