# Supplementary material for: Defective viral genomes of porcine reproductive and respiratory syndrome virus type 2: Generation, characterization and interference effects on viral replication
Source: PLoS Pathog. 2026 Oct 28;22(10):e1014755. doi: 10.1371/journal.ppat.1014755 (PMC13734580; doi:10.1371/journal.ppat.1014755)
Supplement: Supplementary file 2 [file ppat.1014755.s011.pdf]

Fig 3G-original uncropped gel

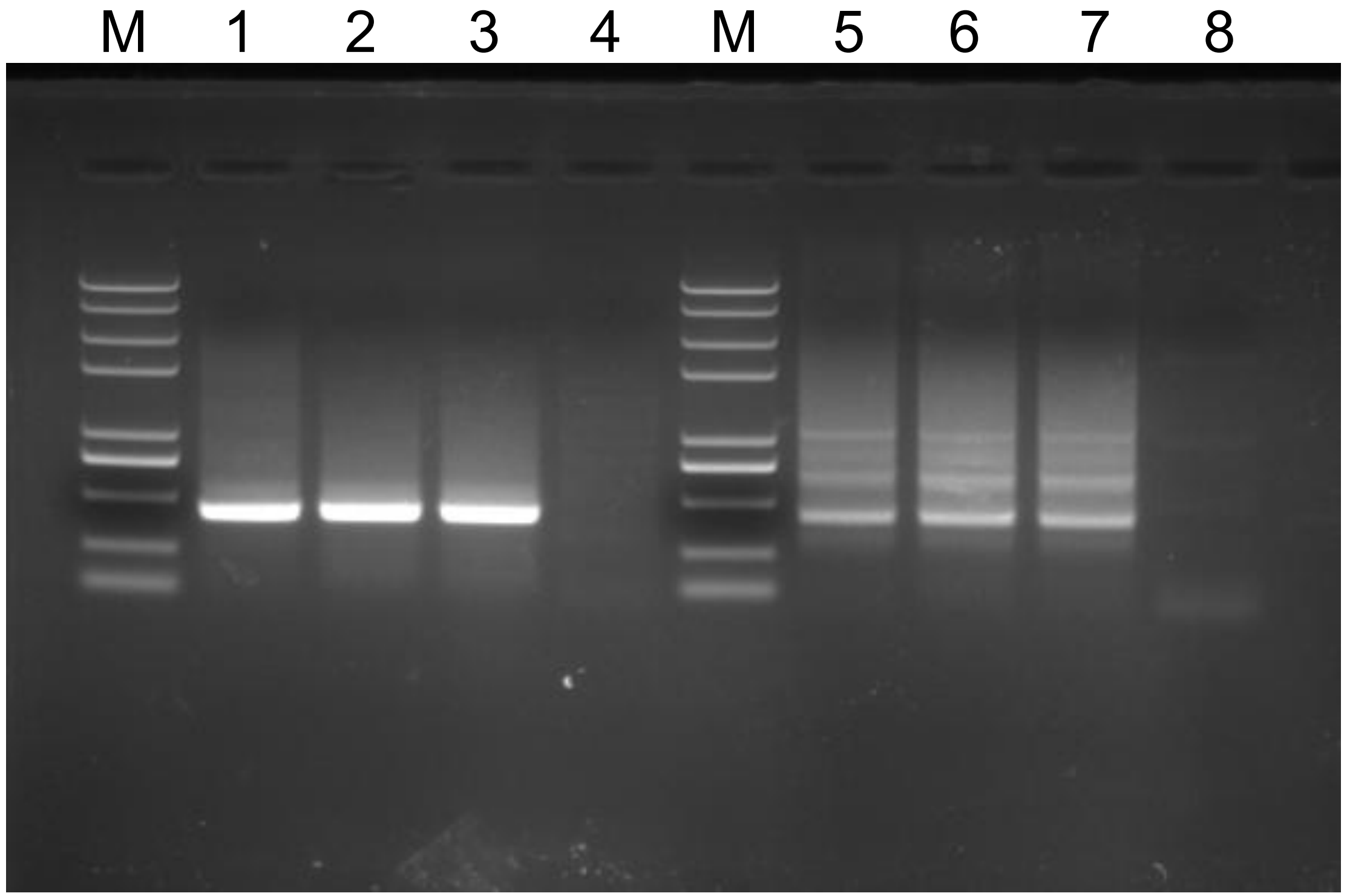

Lane 1: input virus stock;
Lane 2: infected-cell supernatant;
Lane 3: infected-cell lysate;
Lane 4: uninfected cells;
Lane 5: input virus stock;
Lane 6: infected-cell supernatant;
Lane 7: infected-cell lysate;
Lane 8: uninfected cells.
