# Supplementary material for: Defective viral genomes of porcine reproductive and respiratory syndrome virus type 2: Generation, characterization and interference effects on viral replication
Source: PLoS Pathog. 2026 Oct 28;22(10):e1014755. doi: 10.1371/journal.ppat.1014755 (PMC13734580; doi:10.1371/journal.ppat.1014755)
Supplement: Supplementary file 3 [file ppat.1014755.s007.docx]

**S3 Table.** **Primers used in this study.**

| Primer names | Primer sequence (5’-3’) | Primer use |
| --- | --- | --- |
| DVG1-F  DVG1-R  DVG1 probe | cgctcccaccgAaagttcag  gccgcgacttacctttagag  FAM-accaatttgGCgttttcc-MGB | RT-qPCR of DVG1 mRNA |
| GP5-F  GP5-R | GAGTTGTGCTTGATGGTTCCG  GCTGTCATTGCAGAAGTCGTC | RT-qPCR of PRRSV-2 GP5 mRNA |
| IFN-β-F  IFN-β-R | ATTGTGGAGAAACTCCTTGCT  AGGTGCAGACTGCTCATGAAT | RT-qPCR of IFN-β in Marc-145 cells |
| ISG15-F  ISG15-R | CAGCACCTACGAGGTACAGC  TCGAAGGTCAGCCAGAACAG | RT-qPCR of ISG15 in Marc-145 cells |
| GAPDH-F  GAPDH-R | TGCTGACGCCCCTATGTTTG  GTGCTAAGCAGTTGGTGGTG | RT-qPCR of GAPDH in Marc-145 cells |
